# Biochar-biofertilizer combinations enhance growth and nutrient uptake in silver maple grown in an urban soil

**DOI:** 10.1371/journal.pone.0288291

**Published:** 2023-07-18

**Authors:** Melanie A. Sifton, Sandy M. Smith, Sean C. Thomas

**Affiliations:** Institute of Forestry and Conservation, University of Toronto, Toronto, ON, Canada; Universiti Putra Malaysia (UPM), MALAYSIA

## Abstract

Declining tree health status due to pollutant impacts and nutrient imbalance is widespread in urban forests; however, chemical fertilizer use is increasingly avoided to reduce eutrophication impacts. Biochar (pyrolyzed organic waste) has been advocated as an alternative soil amendment, but biochar alone generally reduces plant N availability. The combination of biochar and either organic forms of N or Plant Growth Promoting Microbes (PGPMs) as biofertilizers may address these challenges. We examined the effects of two wood biochar types with *Bacillus velezensis* and an inactivated yeast (IY) biofertilizer in a three-month factorial greenhouse experiment with *Acer saccharinum* L. (silver maple) saplings grown in a representative urban soil. All treatments combining biochars with biofertilizers significantly increased sapling growth, with up to a 91% increase in biomass relative to controls. Growth and physiological responses were closely related to nutrient uptake patterns, with nutrient vector analyses indicating that combined biochar and biofertilizer treatments effectively addressed nutrient limitations of both macronutrients (N, P, K, Mg, Ca), and micronutrients (B, Fe, Mn, Mo, Na, S, and Zn). Biochar-biofertilizer treatments also reduced foliar concentrations of Cu, suggesting potential to mitigate toxic metal impacts common in urban forestry. We conclude that selected combinations of biochar and biofertilizers have substantial promise to address common soil limitations to tree performance in urban settings.

## Introduction

Urban forests have been determined to provide important ecosystem services [1–3], but urban trees often show poor growth and reduced survivorship due to soil limitations [2,4]. Urban soil pollutants have been a concern over the past few decades [5], but other limitations to urban plant-soil interactions remain scarcely investigated despite the urgent need for cost-effective landscape restoration and revegetation techniques [6,7]. Soil factors contributing to reduced vigor of urban trees include soil stripping, mixing, compaction, elevated pH, low moisture retention, and chemical imbalances [8]. As these soil conditions hinder revegetation efforts and often result in critical limitations to soil nutrient and water availability in urban plantings [9], new environmentally-friendly approaches to urban soil and vegetation restoration must be evaluated.

Biochar, a charcoal soil amendment created by pyrolysis of organic material at low oxygen levels, has recently been promoted for use in urban forestry [10,11]. Biochar’s benefits include its resistance to decomposition, resulting in stable and long-lived carbon capture, provision and retention of plant nutrients, liming or stabilization of soil pH, increased soil microbial activity, enhanced moisture retention and availability, and immobilization of toxic substances such as heavy metals [12,13]. While biochar applications have been studied extensively in an agricultural context, research into the potential use of biochar for urban tree applications is still limited to a few studies (eg. [10,14–19]. With municipal governments increasingly restricting use of fertilizers and inorganic materials in the urban landscape [20], there have been calls for innovations in soil amendments for urban ecosystems [21,22]. While significant inroads have been made into sustainable agriculture methods including biofertilizers and organic soil amendments, work has still been limited on how these may be expanded and applied for urban forestry and horticulture [23].

Two potential factors that might contraindicate biochar use in urban forestry are its typically high pH [24], and its potential for erosion by wind and water [25]. Most high temperature biochars are moderately to strongly basic, and studies have documented a liming effect from the addition of higher pH biochars to soils with low pH [26–28]. However, studies examining the impact of biochar on alkaline and human-altered soils common in many urban areas are few [16,29]. Through selection of appropriate feedstock and pyrolysis conditions, it is possible to produce biochars that are near neutral in pH [28,30]. Wind and water transport of biochar can potentially be mitigated by granulation or pelletization of biochars [31,32]. Pelletized or granulated biochars have been investigated principally for planting applications in nursery culture as a substitute for peat, perlite, or vermiculite [33,34], or as a green roof substrate amendment [32,35]. Additions of granulated biochar to fine-textured soils might be expected to differ from effects of conventional biochar mainly due to particle size: the larger-sized biochar granules may specifically increase growth responses by reducing soil bulk density and increasing soil moisture and aeration [35,36].

Existing data on biochar particle size effects on plant growth is limited, yet there is evidence for large differences in species responses [35]. When added to the same sand substrate, velvetleaf growth was enhanced by a biochar with small (0.0635–0.5 mm) particles, while annual ryegrass showed positive growth response by the same biochar with larger particles (2–4 mm) [35]. Biochars used for soil amendment tend to range in size between <0.10 and 4+ mm, but biochars with particles sized at an intermediate range of ~0.5–1 mm have been suggested as optimal based on a recent meta-analysis of plant responses [31].

While biochar is touted as having wide applicability, an additional persistent problem is biochar’s tendency to bind available N forms (in particular ammonium) and thus induce N deficiencies in some plants [37–39]. Ammonium (NH_4_^+^) is primarily bound to biochar via electrostatic adsorption to oxidized surface moieties [40]. A 2019 meta-analysis of biochar effects on soil N availability in agricultural systems found that biochars reduced NH_4_^+^ by a mean of 11%, but that this response varied with environmental conditions [41]. While landscapes with excess N could benefit from biochar’s ability to bind N, plants requiring higher N levels may suffer [42,43]. A review of N use efficiency results concluded that the N immobilization effect of wood biochar was a factor reducing crop yield in the first year after application, but that using lower-temperature high-organic-matter biochars or combining high temperature wood biochar with fertilizer can overcome this problem [39]. A study on biochar effects on nutrients in rangeland soils showed improved N availability and retention when amendments included manure [44], and a meta-analysis of 124 published peer-reviewed biochar agricultural trials across the globe showed that combining biochar with organic fertilizers generally improves N availability [41].

Recent research indicates the potential of Plant Growth Promoting Microorganisms (PGPMs), also referred to as “biofertilizers”, for enhancing nutrient availability, plant disease resistance, and soil moisture availability [45–48]. PGPMs have shown particular promise in combination with biochar in agricultural applications [49,50]. Combining biochar with PGPMs has been demonstrated to be of benefit to plants under drought stress, such as French beans [51], cucumber [52], maize [53], soybeans [54], and a range of other crops [55]. Prior research has also been conducted on the combination of biochar with compost [56,57], compost tea [58], and sewage sludge [10], which facilitate broad-spectrum microbial inoculation of biochar. The use of wood biochars as beneficial soil bacteria carriers has been examined for *Enterobacter* [59], *Pseudomonas* [60], and a *Pseudomonas*, *Serratia*, and *Kosakonia* sp. complex [61], but the focus of biochar inoculation is often on remediation of soil contaminants such as heavy metals [62–64] and pesticides [65]. In one of the few such studies related to trees, biochar was shown to increase soil bacterial diversity, moisture, potassium, and nitrate when combined with the PGPM *Bacillus megaterium* in a sub-tropical eucalyptus plantation [66], but effects on tree growth and physiological performance were not studied directly.

Given the evidence for enhancement of agricultural crops with co-amendments of biochar and either PGPM or organic N forms, we predicted that similar combinations would increase N availability to support a nitrogen-demanding tree species grown on a representative urban soil. We specifically hypothesized that wood-based biochar soil amendments, combined with the beneficial soil microbe *Bacillus velezensis* or an inactivated yeast organic biofertilizer, would increase growth and physiological performance of silver maple saplings grown in a Human-Altered Human-Transported (HAHT) urban neutral-alkaline loam soil. It was expected that biochar effects on maple saplings would be consistent with increased plant nutrient uptake, and that the lower pH of the granulated conifer biochar would better boost growth and physiological performance in saplings. This trial is the first we are aware of to test biochars with these bacterial and yeast-based soil amendment combinations, and the first to examine tree growth and nutrient uptake alongside soil effects for the purpose of urban forestry applications.

## Materials and methods

### Study species and experimental design

*Acer saccharinum* (silver maple) saplings were the target tree species in this greenhouse pot trial. This species was selected for its indeterminate growth habit, propensity toward high fertility soils, common urban forestry use, and because it is native to a wide range of habitats in eastern and central North America [67]. We used one-year-old seed-grown bare-root *A*. *saccharinum* saplings grown from locally-sourced seed (Ferguson Tree Nursery, Kemptville, ON, Canada). The bare-root saplings arrived with a height range of 14–50.3 cm (mean 25.59, SD = 6.96), and a caliper at base diameter range of 3.78–9.74 mm (mean 6.55, SD = 1.32). The trees were sorted by size and randomly allocated to treatment groups so that initial mean tree size distribution was statistically non-significant across groups (ANOVA, p > 0.05).

The soil used was an anthropogenic calcareous loam topsoil stripped from a construction site in Whitby, Ontario, then transported, screened to 1.25 cm, and bulk stored by the supplier (EarthCo Soils, Toronto, ON, Canada). This soil had a pH of 7.1 and was chosen because it is a common regional example of a low-cost Human-Altered or Human-Transported (HAHT) soil moved and reused in urban development [68,69].

Soil amendment treatments consisted of two biochars and two biofertilizers applied to trial pots using a 3 x 3 factorial design. The list of treatments is as follows: 1) control (urban soil), 2) granulated conifer biochar, 3) sugar maple biochar, 4) inactivated yeast (IY), 5) IY with granulated conifer biochar, 6) IY with sugar maple biochar, 7) IY and live *B*. *velezensis* (*Bv*), 8) IY and live *B*. *velezensis* with granulated conifer biochar, and 9) IY and live *B*. *velezensis* with sugar maple biochar. The experiment tested the combination of *B*. *velezensis* with biochar, but a full factorial design was not possible because the *Bv* strain was not available in a registered biofertilizer formulation in Canada without the inactivated yeast.

Biochar amendments were each bulk-mixed by hand at a rate of 40 g of biochar per 2500 ml of soil then added to ~4-L nursery pots, which was equivalent by weight per area to ~20 t/ha. Several prior studies have indicated that 20 t/ha is an appropriate biochar application rate to benefit trees [16,70]. Biofertilizer treatments were mixed using filtered water according to supplier recommendations at a rate of ~0.5% solution for *Bv* with IY and a ~0.56% solution for just IY, and were both applied one week after planting and then again 1 month later.

The experiment was run for three months from early May to August 2018 at a greenhouse in Toronto, Canada where the air temperature ranged from 7.5–40.6°C with a mean of 23.4°C (+- 0.1), without supplemental fertilizer beyond the treatments, nor lighting. Filtered irrigation water was applied in equal amounts to all pots at least every 2–3 days. All pots were randomized and re-spaced biweekly. Treatments were replicated with 13 pots each planted with one maple sapling (*n* = 13, 117 total experimental units).

### Soil amendments

High temperature wood-feedstock biochars created from typical Canadian timber forestry by-products were selected as soil amendments because of their availability and potential for waste stream reuse. The two biochars used in the trial were 1) a mixed conifer biochar made by slow pyrolysis at ~700°C and drum granulated using a proprietary binder [32]; and 2) a loose sugar maple (*Acer saccharum* L.) sawdust biochar created using slow pyrolysis at ~700°C (~10 min. residence time) supplied by Haliburton Biochar Ltd., Haliburton, ON, Canada. The granulated conifer biochar was selected for this study to compare against unbound hardwood biochar because concerns have started to arise about small-particle biochars [25], and also as a means of comparing sapling and soil responses to high-temperature biochars created from softwood vs hardwood feedstocks. As this study shared a control with and ran parallel to another trial, additional urban soil and biochar preparation, characterization methods, and details are provided in Sifton et al. [19].

Plant Growth Promoting Microbes (PGPMs) were selected as biofertilizers to combine with biochars for this urban forestry study due to their availability as soil inoculants and proven plant growth and health benefits in agriculture [45,46]. IY+ *Bv* and IY by itself were both selected from a range of available biofertilizer products based on the prediction that they should result in enhanced N availability with biochars [71] and also because *Bv* should remain viable in slightly alkaline clay and loam urban soils typical of the Greater Toronto Area [72,73]. The IY biofertilizer formulation (trade name Bioreveil®) and the *Bv* + IY formulation (trade name LALRISE VITA®) used in this trial were supplied by Lallemand Inc. (Sault Ste-Marie, ON, Canada).

*Bacillus velezensis* 30322 (*Bv*) is a well-characterized and commercially-available strain of a bacteria that occurs naturally in soil and has been found to enhance phosphorus and nitrogen availability [74], reduce the need for crop fertilization [75], and out-compete plant pathogens in a variety of systems [76–78]. *Bv* used in this trial has been recently reclassified and was formerly known by the synonym *Bacillus amyloliquefaciens*, or as a strain of *B*. *subtilis* [79–81].

While various forms of inactivated *S*. *cerevisiae* have been explored for wine grape production [82,83], as well as for animal feed [84], research is broadening to examine potential applications of IY as a biofertilizer for a broader range of plants. *S*. *cerevisiae*, whether live or dead, has been shown to enhance the growth and health of agricultural crops [85,86], and also has capacity for toxic metal remediation [87,88]. In the present study, IY refers to *Saccharomyces cerevisiae* (Meyen ex E.C. Hansen, 1883) that has been inactivated by thermal treatment. IY is inexpensive to manipulate and produce, is commonly available as a by-product of the food and beverage industries [89], and has received recent attention for industrial and agricultural applications [87,90,91]. IY may be particularly suitable as an organic N source in urban environments due to low cost, ease of use, and lack of adverse odor.

### Data collection

pH and electroconductivity (EC) of biofertilizers, biochars, and soil were analyzed in solution after 24 hrs at 80 rpm on an orbital shaker using an IQ Scientific pH meter and Thermo Scientific Orion Star A112 EC meter, respectively. Biofertilizers were analyzed for plant nutrients using ICP-OES for total P, K, Mg, Ca, Cu, Al, B, Fe, Mn, Mo, Na, S, (Actlabs, Ancaster, ON, Canada) and combustion analysis to determine total C and N (CN628 Elemental Analyzer, LECO Instruments, Mississauga, ON, Canada). Final soil measurements included measures of at least 3 replicates per treatment of soil pH, EC, total carbon and nitrogen (CN628 Elemental Analyzer, LECO Instruments, Mississauga, ON, Canada), and soil moisture (SM150 Dynamax Inc. Houston TX, USA) after a 3-day dry down period.

Tree growth was measured by initial and final sapling height and stem diameter at base (caliper), as well as intermediate and final leaf area (LI-3100 optical area meter, Licor Biosciences, Lincoln, NB, USA). Final sapling dry mass of below-ground and above-ground (stem + foliage) parts were determined after drying to constant dry mass at 60°C in a forced-air oven, and combined for final biomass.

Sapling physiological measurements included leaf chlorophyll content (CCI) using a CCM-200plus (Opti-Sciences Inc., Hudson, NH, USA), leaf chlorophyll fluorescence (Fv/Fm) via saturated pulse method with a Mini-Pam fluorometer (Heinz Walz GmbH, Effeltrich, Germany), and leaf gas-exchange, including (A_sat_), stomatal conductance (g_s_), and instantaneous water-use efficiency (WUE). The latter measurements were made with an LI-6400 XT photosynthesis system (Licor Biosciences, Lincoln, NE, USA) on fully-expanded leaves in the upper crown at 1000 μmol m^2^s^-1^ PAR, 400 ppm CO_2_, leaf temperatures 20–25°C, and humidity of 45–60%. Additional details on data collection methods, particularly pertaining to soil attributes, sapling growth, and ecophysiological measurements, are described in Sifton et al. (2022) [19].

Leaf sample tissues were dried at 60°C, ground, and subset into 3 reps each containing the same amount of materials from 4 replicates so that *n* = 3 for all results. Sapling foliar elemental content analyses included total C, N (combustion method using CN628 Elemental Analyzer, LECO Instruments, Mississauga, ON, Canada); N thermal conductivity detection (Elementar Vario Cube); and total P, K, Mg, Ca, Cu, Al, B, Fe, Mn, Mo, Na, S, and Zn (microwave digestion and ICP-OES method using Agilent 5110, Agriculture and Food Laboratory, University of Guelph, ON, Canada).

### Statistical analysis

R version 3.6.2 was used to carry out statistical analyses [92]. Biofertilizer and biochar type were analyzed as main effects using 1- and 2-way ANOVA (analysis of variance) conducted on untransformed response variable data. ANOVA assumptions were assessed using graphical analyses; treatment with means that were significantly different from the control were determined using Dunnett’s post-hoc tests. Bare-root saplings were allocated randomly to treatments groups at the start of the experiment, and no statistically significant differences in starting height were detected between groups. ANCOVA (analysis of co-variance) further confirmed that initial height was not a contributing factor in final leaf area, nor final height results, but may have contributed somewhat to final sapling dry mass; results for ANOVA without initial size as a covariate are presented. Vector analyses were used to interpret shifts in leaf nutrients induced by treatments [93,94]. Vector diagram means were calculated for each sapling using leaf dry mass and mean concentration of nutrients.

## Results

### Soil, biochar, and biofertilizer properties

The HAHT urban soil began with a mean pH of 7.1, while the sugar maple and mixed granulated conifer biochars had an initial mean pH of 7.87 and 6.54, respectively (Table 1). Further details on the soil and biochar material characterizations for this experiment can be found in [19]. As a predictable consequence of the addition of a granulation binder in one of the biochars, the CN ratio was over 4 times higher in the unbound sugar maple biochar (182.07) compared to the granulated conifer biochar (42.67), and the latter was higher in N (Table 1). The two biochars contained similar amounts of P and K, but the sugar maple biochar was higher in Mg while the conifer biochar was higher in Ca. Biochar characterizations showed that the granulated conifer biochar tended to contain higher levels of most micronutrients, with B, Mn, Rb, and Sr as exceptions. The IY and IY + *Bv* biofertilizers had similar pH with a mean of 6.20 and 6.14, respectively (Table 1). IY + *Bv* had EC values far higher than just IY alone. Biofertilizer nutrient characterizations showed that both had similar N, P, Ca, Mg contents, while IY alone was higher in S and Fe, and IY + *Bv* was notably higher in K, B, Cu, and Mn.

**Table 1:** Characteristics of biochars, biofertilizers, and soil.

Attribute	Sugar maple biochar	Conifer granulated biochar	IY (*S*. *cerevisiae*)	IY + *Bv* (*B*. *velezensis*)	Urban soil
pH (H_2_O)	7.87 (0.05)	6.54 (0.40)	6.20 (0.01)	6.14 (0.01)	7.10 (0.01)
Electrical Conductivity (μS cm^-1^)	62.73 (3.46)	76.80 (1.91)	1027 (88.04)	1895 (219.20)	458.00 (7.55)
Total C (%)	78.29 (0.30)	60.84 (0.14)	43.49 (0.01)	40.24 (0.03	3.11 (0.12)
N (%)	0.43 (0.00)	1.46 (0.00)	7.49 (0.01)	8.68 (0.08)	0.13 (0.01)
C:N ratio	182.07	41.67	5.81	4.64	23.92
P (%)	0.03 (0.00)	0.05 (0.00)	1.21 (0.00)	1.08 (0.01)	< 0.01
K (%)	0.40 (0.03)	0.30 (0.01)	1.74 (0.00)	3.04 (0.04)	0.01
Ca (%)	0.88 (0.10)	1.16 (0.01)	0.17 (0.00)	0.15 (0.00)	0.46
Mg (%)	0.13 (0.01)	0.24 (0.00)	0.16 (0.00)	0.13 (0.00)	0.01
Na (%)	0.03 (0.00)	0.09 (0.01)	ND	ND	ND
S (%)	<1 (0.00)	<1 (0.00)	0.36 (0.00)	0.01 (0.01)	ND
Fe (ppm)	0.23 (0.04)	0.73 (0.02)	73.00 (0.27)	39.00 (0.27)	118.00
B (ppm)	ND	ND	1.00 (0.00)	2.00 (0.00)	ND
Cu (ppm)	6.83 (1.07)	54.1 (14.36)	5.00 (0.00)	10.00 (0.27)	1.40
Mn (ppm)	595.33 (54.12)	273.00 (3.61)	8.00 (0.27)	14.00 (0.27)	22.90
Mo (ppm)	0.97 (0.62)	3.03 (0.07)	1.00 (0.00)	1.00 (0.00)	ND
Al (ppm)	900 (100)	5367 (200)	4.00 (0.27)	5.00 (0.00)	ND
Zn (ppm)	103.33 (11.05)	141.00 (50.95)	ND	ND	2.80

Values are means (±SE) of triplicate measurements, with the exception of topsoil nutrients which were provided by SGS Laboratories, Guelph, ON, Canada. Biofertilizer nutrients were measured via combustion method for C and N and ICP-OES for other elements. pH and EC of soil was measured after 24 hr on an orbital shaker using 1:3 (v:v) ratio of soil to de-ionized water; biochar and biofertilizers were measured at a 1:20 ratio.

### Soil responses

**Fig 1:**
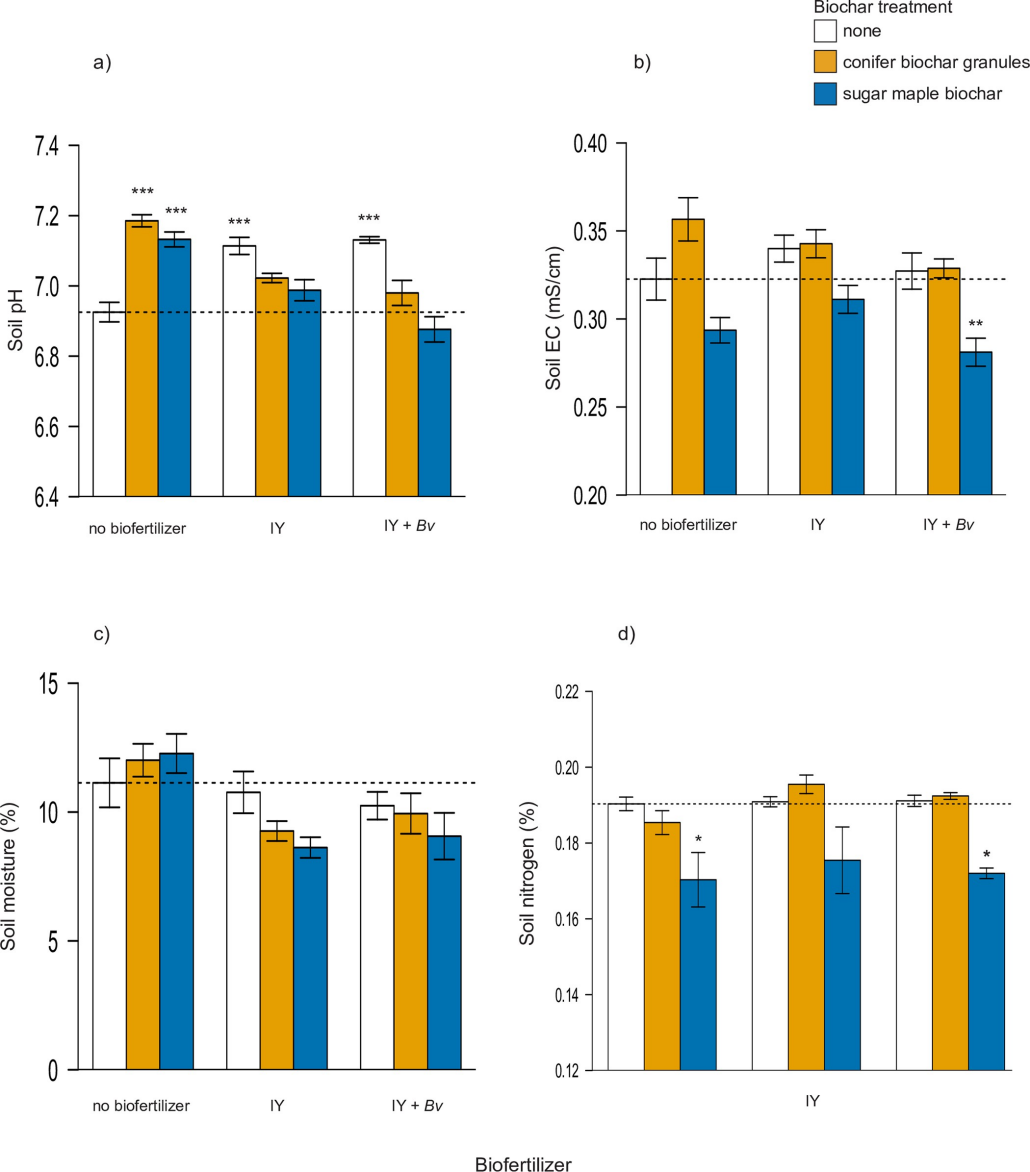
Mean measures of final soil a) pH, b) EC, c) moisture, d) total N. Statistically significant differences between control and treatment groups are indicated by asterisks (1-Way ANOVA, Dunnett’s test, * = p < 0.05, ** = p < 0.01, *** = p < 0.001). N = 13 trees for all treatments. EC = electrical conductivity, IY = inactivated yeast, *Bv* = *Bacillus velezensis*. Dashed line indicates control mean.

Soil pH increased with additions of biochars and biofertilizers applied alone (Table 2; Dunnett, p < 0.05), but no significant changes were observed compared to the control when biochars and biofertilizers were combined (Fig 1A). A 2-way ANOVA showed that biochar had a significant effect on soil EC as well (Table 2). All treatments with sugar maple biochar reduced final soil EC compared to the control, but only sugar maple biochar combined with IY + *Bv* was significantly lower than the control (-13%) (Table 2; Fig 1B). When measured after a dry-down period at the end of the experiment, the treatments had no significant effect on soil moisture (Fig 1C). Soil N decreased as much as 10% with the addition of sugar maple biochar, but these soil N changes were barely significant compared to the control (Table 2; Fig 1D). Final soil C in all sugar maple biochar treatments was 85–87% higher than control soil (Dunnett, p < 0.001), while significant changes in soil C were not detected in any other treatment (S1 Fig in S1 File). Similarly, soil C:N ratios were consistently double in treatments containing sugar maple biochar compared to the control or other treatments (Figs 1D and S1 Fig in S1 File).

**Table 2:** 2-WAY ANOVA results of treatment effects on soil.

	Deg. Freedom	Sum of Squares	Mean Square	F Statistic	p-value
Soil pH					
Biochar	2	0.100	0.050	6.042	**0.003**
Biofertilizer	2	0.139	0.070	8.400	**<0.001**
Biochar x biofertilizer	4	0.927	0.232	27.962	**<0.001**
*Error*	106	0.878	0.008		
Soil EC					
Biochar	2	0.047	0.023	22.362	**<0.001**
Biofertilizer	2	0.007	0.003	3.273	**0.042**
Biochar x biofertilizer	4	0.006	0.001	1.389	0.243
*Error*	107	0.112	0.001		
Soil moisture					
Biochar	2	10.400	5.210	0.786	0.458
Biofertilizer	2	121.600	60.790	9.181	**<0.001**
Biochar x biofertilizer	4	40.100	10.020	1.514	0.203
*Error*	108	715.000	6.620		
Soil nitrogen					
Biochar	2	0.002	0.001	19.635	**<0.001**
Biofertilizer	2	<0.001	<0.001	1.228	0.316
Biochar x biofertilizer	4	<0.001	<0.001	0.372	0.826
*Error*	18	0.001	<0.001		
Soil carbon					
Biochar	2	35.370	17.683	453.861	**<0.001**
Biofertilizer	2	0.010	0.003	0.068	0.934
Biochar x biofertilizer	4	0.010	0.002	0.046	0.996
*Error*	18	0.700	0.039		

Significant results (p < 0.05) are bold.

### Sapling growth responses

**Fig 2:**
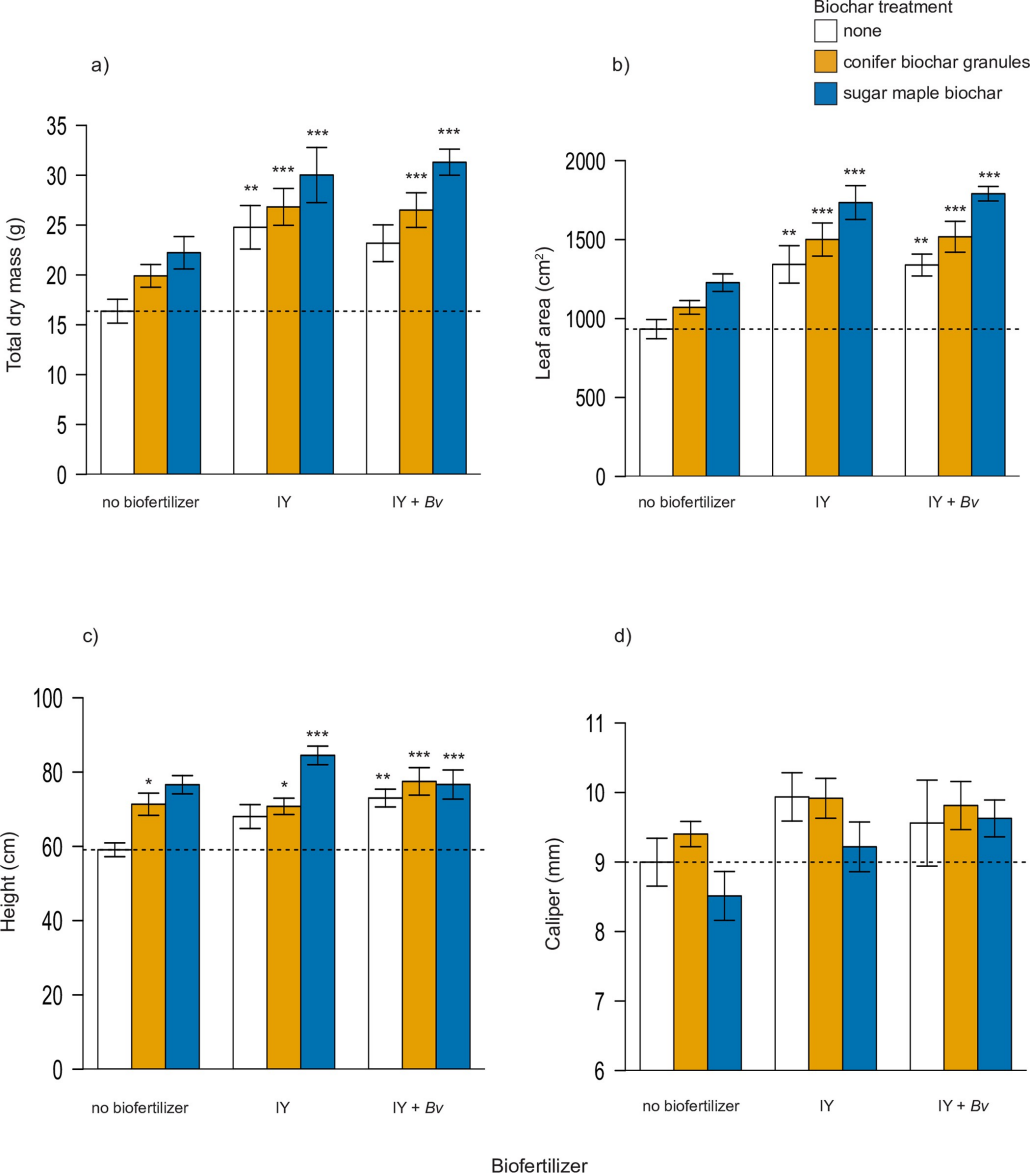
Tree responses in mean final a) total dry mass, b) leaf area, c) height, d) caliper. Statistically significant differences between control and treatment groups are indicated by asterisks (1-Way ANOVA, Dunnett’s test, * = p < 0.05, ** = p < 0.01, *** = p < 0.001). Dashed line indicates control mean. N = 13 trees for all treatments. IY = inactivated yeast, *Bv* = *Bacillus velezensis*.

Final maple sapling total dry mass was significantly higher than the control group (Table 3; Dunnett, p < 0.001) for all treatments where biochar was combined with a biofertilizer, as well as with IY alone (Fig 2A). The highest increase in dry mass compared to the control occurred when sugar maple biochar was combined with IY (+91%), followed by sugar maple biochar combined with *Bv* + IY (+83%). Final sapling leaf area was also significantly higher than the control group for all treatments with biofertilizers, with comparable increases also measured when sugar maple biochar was combined with IY (+92%), and sugar maple biochar combined with *Bv* + IY (+86%) (Table 3; Fig 2B). For final sapling height, all treatments with biochars alone or in combination with biofertilizers were significantly taller than the control. IY treatment alone did not result in significant increases in height, but IY+*Bv* had a mean final tree height which was 24% greater than the control, while IY added to pots with sugar maple biochar increased maple height by 43% over the control (Table 3; Fig 2C). No statistically significant differences were detected for final sapling diameter at base among treatment groups compared to the control (Table 3; Fig 2D).

**Table 3:** 2-way ANOVA results of final sapling growth, allocation, and ecophysiological responses.

	Deg. Freedom	Sum of Squares	Mean Square	F Statistic	p-value
Total dry mass					
Biochar	2	803.000	401.400	9.471	**<0.001**
Biofertilizer	2	1503.000	751.600	17.736	**<0.001**
Biochar x biofertilizer	4	39.000	9.800	0.232	0.920
*Error*	108	4577.000	42.400		
Leaf area					
Biochar	2	2826023.000	1413012.000	15.838	**<0.001**
Biofertilizer	2	5523938.000	2761969.000	30.959	**<0.001**
Biochar x biofertilizer	4	88376.000	22094.000	0.248	0.911
*Error*	108	9635142.000	89214.000		
Height					
Biochar	2	3067.000	1533.600	14.190	**<0.001**
Biofertilizer	2	990.000	494.900	4.580	**0.012**
Biochar x biofertilizer	4	1206.000	301.500	2.790	**0.030**
*Error*	108	11670.000	108.100		
Caliper					
Biochar	2	7.000	3.498	2.054	0.133
Biofertilizer	2	13.050	6.523	3.830	**0.025**
Biochar x biofertilizer	4	2.960	0.741	0.435	0.783
*Error*	108	183.940	1.703		
Root fraction					
Biochar	2	0.006	0.003	1.193	0.307
Biofertilizer	2	0.010	0.005	1.832	0.165
Biochar x biofertilizer	4	0.013	0.003	1.178	0.324
*Error*	108	0.288	0.003		
Height:caliper ratio					
Biochar	2	5868.000	2933.800	13.437	**<0.001**
Biofertilizer	2	85.000	42.300	0.194	0.824
Biochar x biofertilizer	4	2943.000	735.800	3.370	**0.012**
*Error*	108	23580.000	218.300		
Leaf area ratio					
Biochar	2	70.000	34.920	0.538	0.585
Biofertilizer	2	67.000	33.370	0.514	0.599
Biochar x biofertilizer	4	220.000	54.880	0.846	0.499
*Error*	108	7007.000	64.880		
Leaf mass:leaf area					
Biochar	2	<0.001	<0.001	0.197	**0.031**
Biofertilizer	2	<0.001	<0.001	3.588	0.821
Biochar x biofertilizer	4	<0.001	<0.001	1.126	0.348
*Error*	108	<0.001	<0.001		
Leaf N concentration					
Biochar	2	0.012	0.006	5.282	**0.006**
Biofertilizer	2	0.125	0.063	53.851	**<0.001**
Biochar x biofertilizer	4	0.003	0.001	0.622	0.648
*Error*	108	0.126	0.001		
Leaf chlorophyll (CCI)					
Biochar	2	14.740	7.371	3.580	**0.0312**
Biofertilizer	2	55.200	27.600	13.404	**<0.001**
Biochar x biofertilizer	4	0.940	0.234	0.114	0.978
*Error*	108	222.390	2.059		
Leaf fluorescence (Fv/Fm)					
Biochar	2	<0.001	<0.001	0.250	0.780
Biofertilizer	2	0.005	0.003	5.780	**0.004**
Biochar x biofertilizer	4	<0.001	<0.001	0.227	0.923
*Error*	108	0.050	<0.001		

Significant results (p < 0.05) are bold.

**Fig 3:**
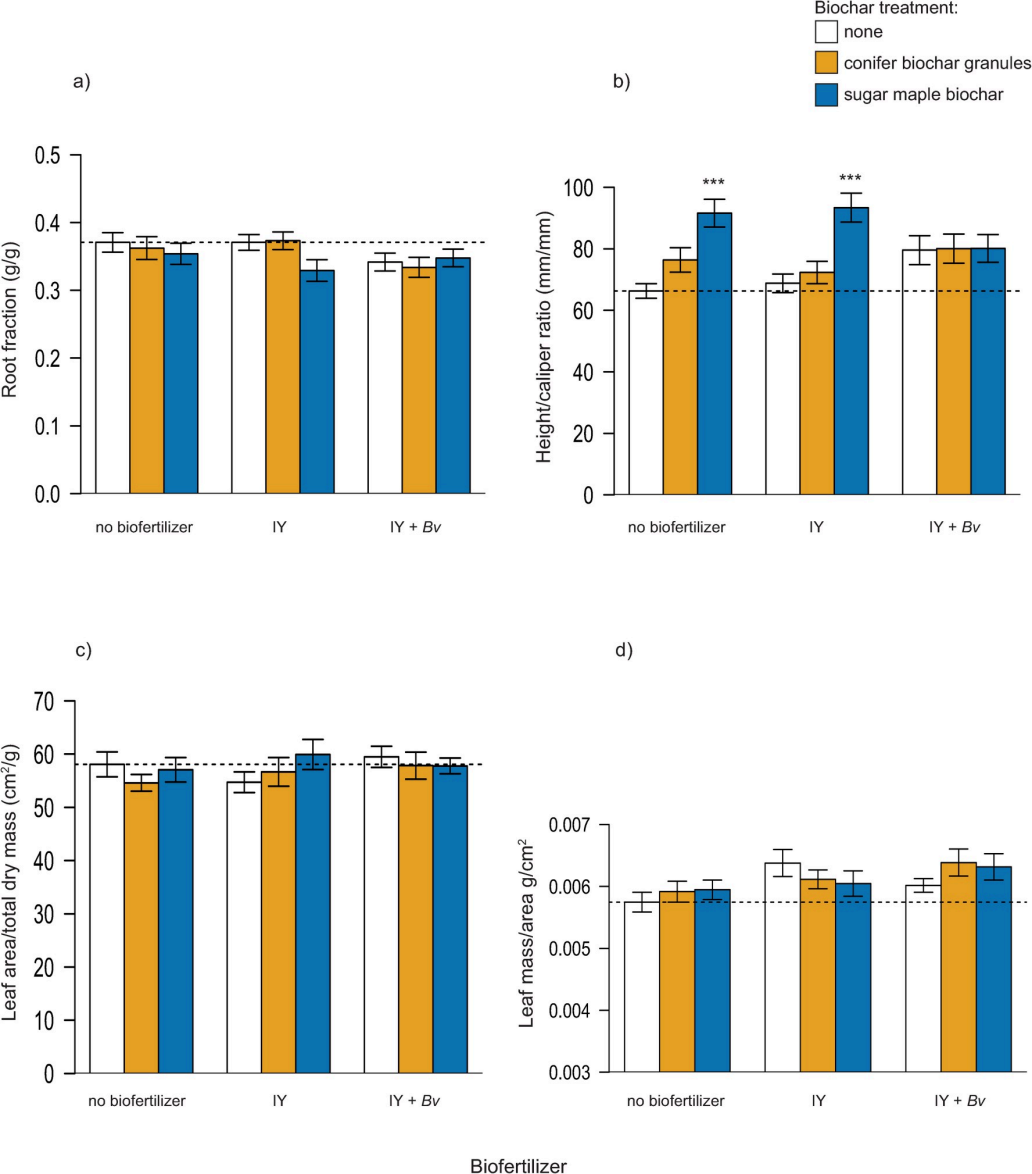
Tree allocation responses a) root fraction of biomass, b) height:caliper ratio, c) leaf area ratio, d) leaf mass per area. Statistically significant differences between the control and treatment groups are indicated by asterisks (1-Way ANOVA, Dunnett’s test, *** = p < 0.001). N = 13 trees for all treatments. Dashed line indicates control mean. IY = inactivated yeast, *Bv* = *Bacillus velezensis*.

Final sapling root fraction (root dry mass/total dry mass) and leaf area ratio showed no significant differences between treatment groups (Table 3; Fig 3A and 3C). Differences in mean height:diameter ratio were most influenced by biochar additions (Table 3): significant increases compared to the control were measured with sugar maple biochar alone (+38%) and sugar maple biochar combined with *Bv* + IY (+41%) (Fig 3B). Microbial inoculation had a significant effect on leaf mass to leaf area ratio (Table 3), though no treatment mean was significantly different than the control (Dunnett, p < 0.05) (Fig 3D).

### Sapling leaf nutrients and vector analyses

**Fig 4:**
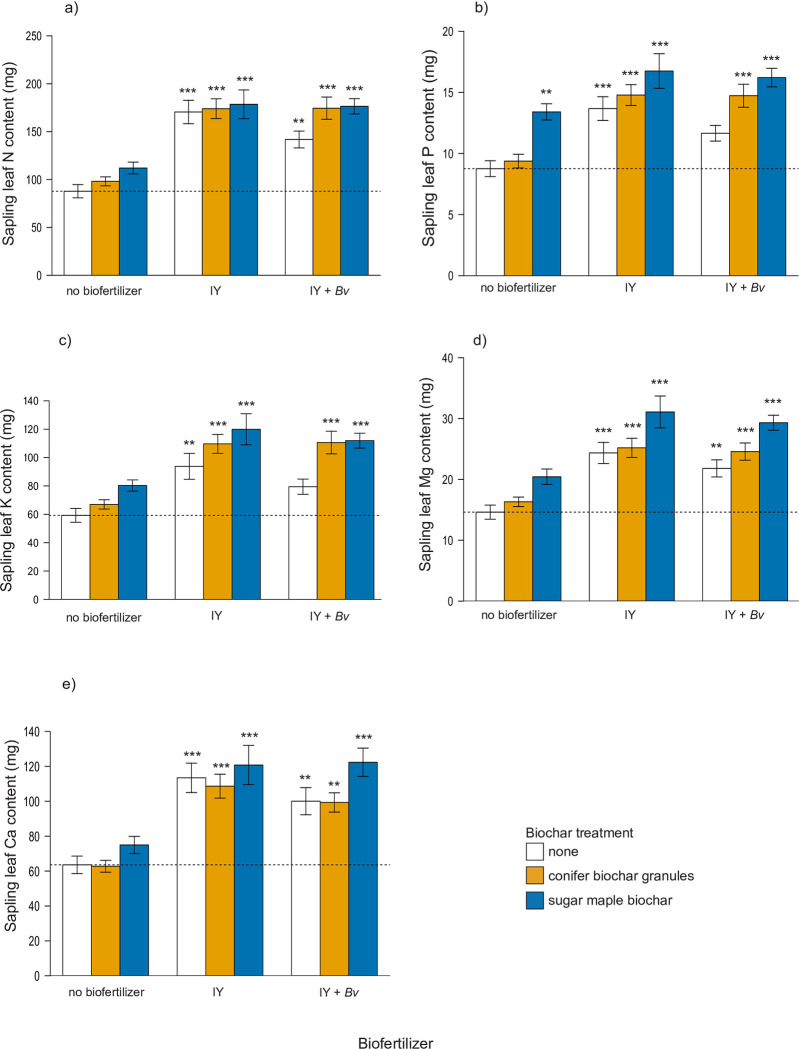
Final sapling leaf macronutrient content (mg) of: a) nitrogen, b) phosphorus, c) potassium, d) magnesium, e) calcium. Statistically significant differences between the control and the treatment groups are indicated by asterisk (1-way ANOVA, Dunnett’s test, ** = p < 0.01, *** = p < 0.001). Dashed line indicates control mean. IY = inactivated yeast, *Bv* = *Bacillus velezensis*.

Leaf N content increased in all treatments compared to the control, and positive responses were highly significant in all treatments with biofertilizers (Fig 4A). Leaf N content more than doubled (+115–125%) in all treatments combining biochar with biofertilizers. Two-way ANOVA also indicated that biochars and biofertilizers had a significant effect on final leaf nitrogen concentration (Table 4).

**Table 4:** 2-way ANOVA results of final sapling leaf nutrient content.

Leaf nutrient Factor	Deg. Freedom	Sum of Squares	Mean Square	F Statistic	p-value
N					
Biochar	2	9388.000	4694.000	4.069	**0.020**
Biofertilizer	2	119358.000	59679.000	51.731	**<0.001**
Biochar x biofertilizer	4	3623.000	906.000	0.785	0.537
*Error*	99	114209.000	1154.000		
P					
Biochar	2	306.800	153.380	17.112	**<0.001**
Biofertilizer	2	421.100	210.540	23.489	**<0.001**
Biochar x biofertilizer	4	34.400	8.590	0.958	0.434
*Error*	99	887.400	8.960		
K					
Biochar	2	13260.000	6630.000	11.936	**<0.001**
Biofertilizer	2	30936.000	15468.000	27.847	**<0.001**
Biochar x biofertilizer	4	1705.000	426.000	0.767	0.549
*Error*	99	54990.000	555.000		
Mg					
Biochar	2	863.600	431.800	15.155	**<0.001**
Biofertilizer	2	1959.500	979.800	34.387	**<0.001**
Biochar x biofertilizer	4	19.700	4.900	0.173	0.952
*Error*	99	2820.700	28.500		
Ca					
Biochar	2	5277.000	2639.000	4.29	**0.016**
Biofertilizer	2	46654.000	23327.000	37.93	**<0.001**
Biochar x biofertilizer	4	835.000	209.000	0.34	0.851
*Error*	99	60892.000	615.000		

Significant results (p < 0.05) are bold.

Biochar and biofertilizer treatments had a significant effect on all leaf macronutrient contents (Table 4 and S5 in S2 File). The final mean leaf content of P, K, Mg, and Ca were all significantly higher in treatments with IY by itself, and all treatments that combined either biochar with either biofertilizer (Dunnett, P <0.05) (Fig 4). The combination of sugar maple biochar with IY generally showed the most increases in leaf macronutrient contents compared to control (N +103%, P +91%, K +102%, Mg +113%). All the treatments containing IY without *Bv* had highly significant increases in leaf macronutrient contents compare to control. The IY + *Bv* treatment without biochar was significantly higher in leaf N, Mg, and Ca content, but not significantly higher than the control in P and K. When analyzed using 1-way ANOVA and Dunnett’s test (p < 0.05), treatment pots containing biochar without any biofertilizers were not significantly higher than the control in the content of any of these macronutrients, with the exception of P, which was significantly higher in the treatment that contained sugar maple biochar by itself.

**Fig 5:**
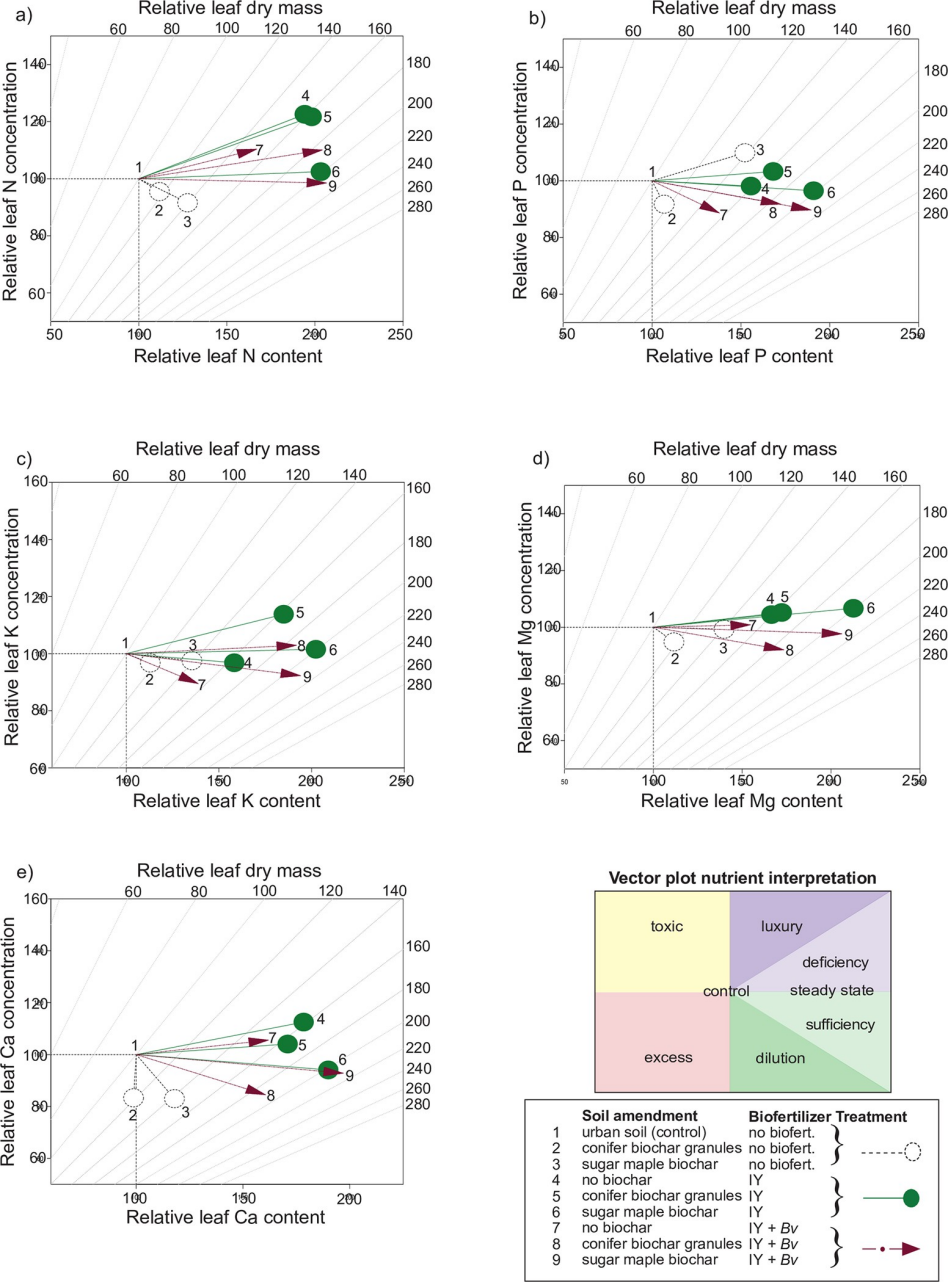
Vector diagrams of relative final leaf tissue macronutrient concentration vs content vs dry mass of: a) nitrogen, b) phosphorus, c) potassium, d) magnesium, e) calcium. IY = inactivated yeast, *Bv* = *Bacillus velezensis*.

Interpretation of final leaf macronutrient shifts compared to the control was conducted using vector diagrams of relative leaf nutrient content (Fig 4) vs concentration (S4 and S7 Figs in S1 File). The vector plot for leaf N shows that the treatments with only biochar had sufficient N but at levels that were diluted due to tree growth (Fig 5A). The two treatments combining sugar maple biochar with biofertilizers were both approaching a steady state of growth with sufficient N content compared to leaf mass. The treatments containing just IY and conifer biochar granules with IY were both heading toward luxury N levels.

The vector plot showing relative P indicates that only sugar maple biochar by itself resulted in deficient phosphorus levels while all IY treatments resulted in close to a steady state of P, and all IY + *Bv* treatments had sufficient P (Fig 5B). The K and Mg vector plots show that all treatments resulted in potassium levels slightly above or below what is required for a steady state of growth (Fig 5C and 5D). The Ca vector indicates calcium levels supportive of mostly steady growth for all treatments with biofertilizers or with biofertilizers combined with biochar, but with an antagonistic level of Ca and limitations on growth with the treatment containing conifer biochar granules (Fig 5E). With all macronutrient vector analyses, the sugar maple biochar combined with either the IY or the IY + *Bv* reached the closest to steady-state leaf nutrient levels with the most increased growth compared to the control.

**Fig 6:**
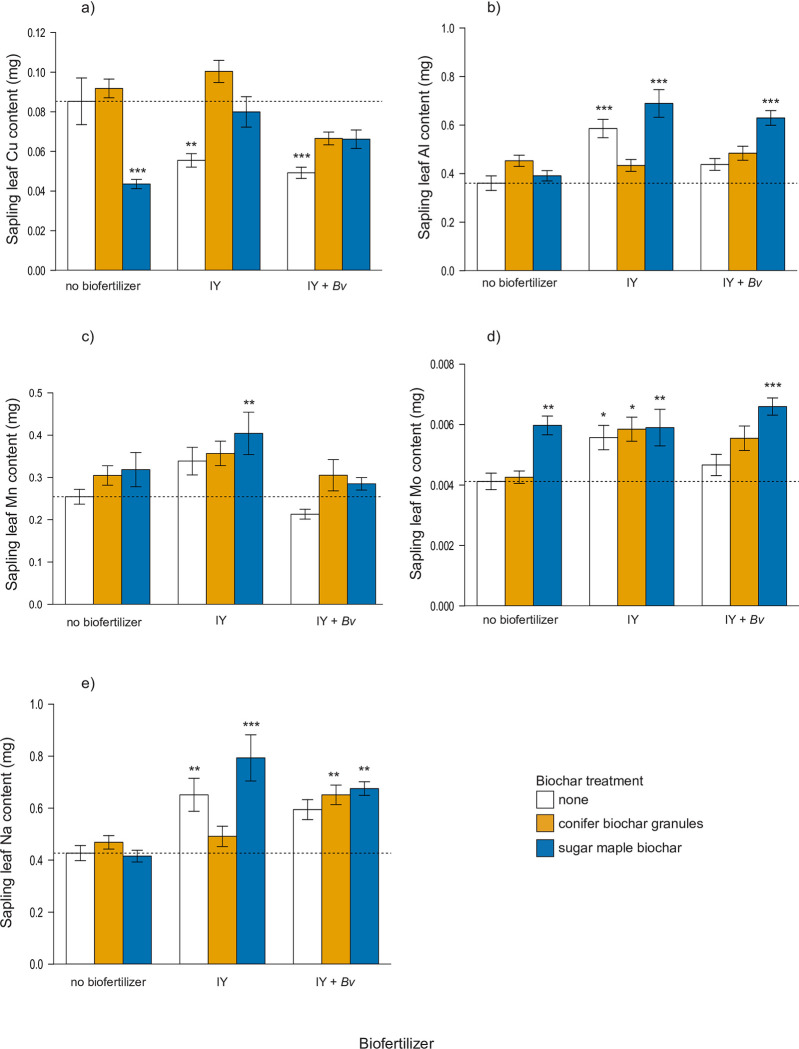
Final sapling leaf micronutrient content (mg) of: a) copper b) aluminum, c) manganese, d) molybdenum, e) sodium. Statistically significant differences between the control and the treatments are indicated by asterisk (1-way ANOVA, Dunnett’s test, * = p < 0.05, ** = p < 0.01, *** = p < 0.001). Dashed line indicates control mean. IY = inactivated yeast, *Bv* = *Bacillus velezensis*.

Analyses using 1-way ANOVA and Dunnett’s test revealed significant (p <0.005) differences in final leaf contents of the micronutrients Cu, Al, Mn, Mo, Na, and Zn (Fig 6), and 2-way ANOVA indicated that all leaf micronutrient contents were influenced by biochar and/or biofertilizer treatments (Table 5). The mean Cu leaf content was significantly reduced in the sugar maple biochar treatment (-49%), as well as in the treatments with just IY (-35%) and IY + *Bv* (-42%) (Fig 6A). In contrast to Cu, Al content increased significantly in the treatments with just IY (+62%), and in the two treatments where IY and IY + *Bv* was added to sugar maple biochar (+91% and +5%, respectively) (Fig 6B). Mn leaf content increased significantly in only one treatment: sugar maple biochar inoculated with IY (+59%) (Fig 6C). Mo increased significantly in all treatments that contained sugar maple biochar (+43–60%), but IY by itself and IY combined with conifer biochar granules were also significantly higher in Mo content compared to the control (Fig 6D). Na leaf content was significantly higher than the control in the IY treatment, as well as in the treatments where IY + *Bv* was added to either biochar, but IY added to sugar maple biochar had the greatest mean increase (+86%) (Fig 6E).

**Table 5:** 2-way ANOVA results of final sapling leaf micronutrient content.

Leaf nutrient Factor	Deg. Freedom	Sum of Squares	Mean Square	F Statistic	p-value
Cu					
Biochar	2	0.013	0.006	15.395	**<0.001**
Biofertilizer	2	0.006	0.003	7.514	**<0.001**
Biochar x biofertilizer	4	0.018	0.005	11.109	**<0.001**
*Error*	99	0.041	<0.001		
Al					
Biochar	2	0.295	0.148	11.678	**<0.001**
Biofertilizer	2	0.532	0.266	21.072	**<0.001**
Biochar x biofertilizer	4	0.395	0.099	7.811	**<0.001**
*Error*	99	1.251	0.013		
B					
Biochar	2	0.2515	0.12577	14.7850	**<0.001**
Biofertilizer	2	0.2981	0.14905	17.5210	**<0.001**
Biochar x biofertilizer	4	0.0463	0.01156	1.3590	0.2540
*Error*	99	0.8422	0.00851		
Fe					
Biochar	2	0.900	0.450	11.968	**<0.001**
Biofertilizer	2	1.561	0.781	20.757	**<0.001**
Biochar x biofertilizer	4	0.417	0.104	2.769	**0.031**
*Error*	99	3.724	0.038		
Mn					
Biochar	2	0.09070	0.04534	3.95000	**0.02237**
Biofertilizer	2	0.19040	0.09518	8.29000	**<0.001**
Biochar x biofertilizer	4	0.02030	0.00507	0.44100	0.77853
*Error*	99	1.13660	0.01148		
Mo					
Biochar	2	0.00004	0.00002	10.46400	**0.00008**
Biofertilizer	2	0.00002	0.00001	5.94000	**0.00366**
Biochar x biofertilizer	4	0.00001	0.00000	1.97600	0.10397
*Error*	99	0.00017	0.00000		
Na					
Biochar	2	0.1638	0.0819	3.2270	**0.0439**
Biofertilizer	2	1.0170	0.5085	20.0370	**<0.001**
Biochar x biofertilizer	4	0.4435	0.1109	4.3690	**0.0027**
*Error*	99	2.5125	0.0254		
S					
Biochar	2	159.0000	79.4800	9.9750	**<0.001**
Biofertilizer	2	408.9000	204.4700	25.6620	**<0.001**
Biochar x biofertilizer	4	32.3000	8.0900	1.0150	**0.4036**
*Error*	99	788.8000	7.9700		
Zn					
Biochar	2	0.336	0.168	14.354	**<0.001**
Biofertilizer	2	0.559	0.280	23.869	**<0.001**
Biochar x biofertilizer	4	0.213	0.053	4.551	**0.002**
*Error*	99	1.160	0.012		

Significant results (p < 0.05) are bold.

**Fig 7:**
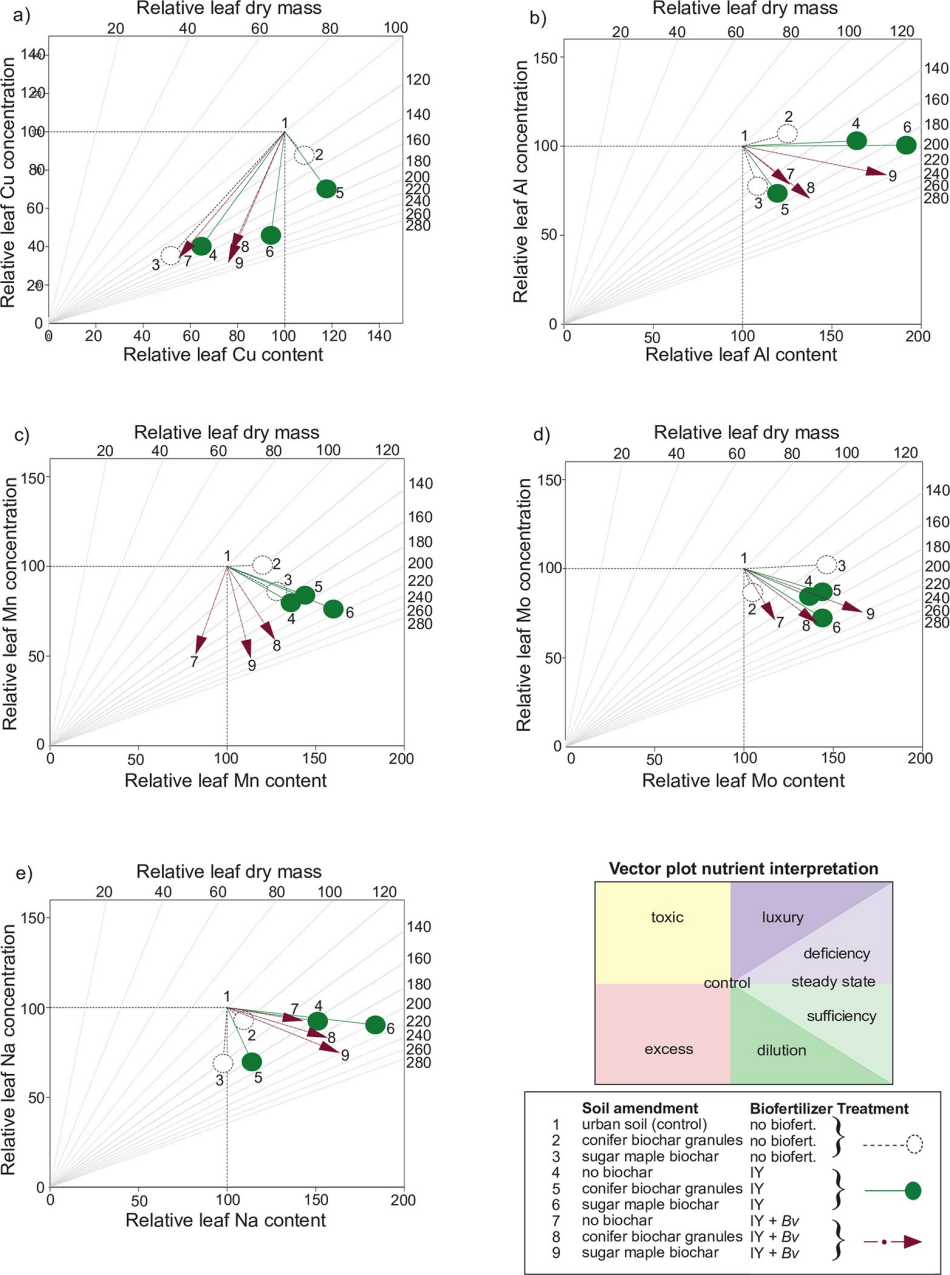
Vector diagrams of relative final leaf tissue micronutrient concentration vs content vs dry mass of: a) copper b) aluminum, c) manganese, d) molybdenum, e) sodium. IY = inactivated yeast, *Bv* = *Bacillus velezensis*.

Vector analysis of leaf copper revealed that, compared to the control, Cu was at antagonistic levels for all treatments except for conifer biochar and conifer biochar combined with IY (Fig 7A). A vector analysis of Al showed that IY alone or combined with sugar maple biochar, as well as sugar maple biochar combined with IY + *Bv* were approaching a steady state balancing this nutrient need with growth (Fig 7B and S5 Fig in S1 File). All treatments analyzed with vector analysis for leaf Mn indicated that manganese was sufficient except for toxic Mn levels induced in the IY + *Bv* treatment. All treatments resulted in steady state or sufficient Mo levels in leaves (Fig 7D). The vector analysis of Na indicated that sugar maple biochar alone was approaching toxic foliar Na levels, but that all the other treatments induced sufficient Na leaf accumulation (Fig 7E).

**Fig 8:**
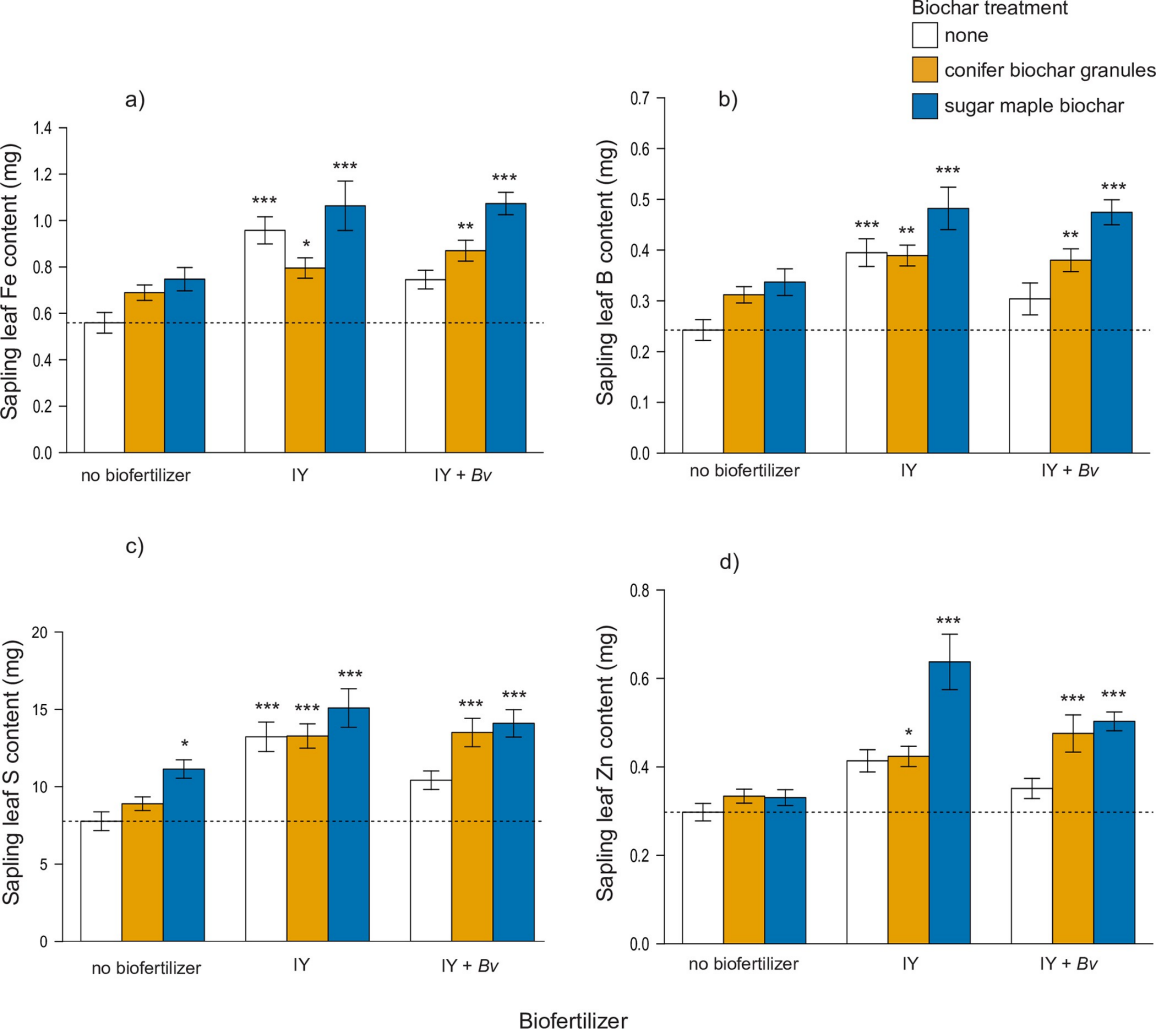
Final sapling leaf micronutrient content (mg) of: a) iron b) boron, c) sulphur, d) zinc. Statistically significant differences between the control and the treatment groups are indicated by asterisk (1-way ANOVA, Dunnett’s test, * = p < 0.05, ** = p < 0.01, *** = p < 0.001). Dashed line indicates control mean. IY = inactivated yeast, *Bv* = *Bacillus velezensis*.

Fe leaf content increased significantly with IY alone (+71%) or when combined with either biochar (Fig 8A), and the biochars combined with either biofertilizer were also significantly higher in Fe compared to the control (+42–92%). The leaf content of micronutrients B and S followed a pattern very similar to that of leaf Fe in regard to which treatments increased significantly compared to the control (Fig 8B and 8C). Zn leaf content only increased significantly compared to the control in treatments that combined biochar with a biofertilizer (Fig 8D). IY combined with sugar maple biochar showed the greatest increase in leaf Zn content (+114%).

**Fig 9:**
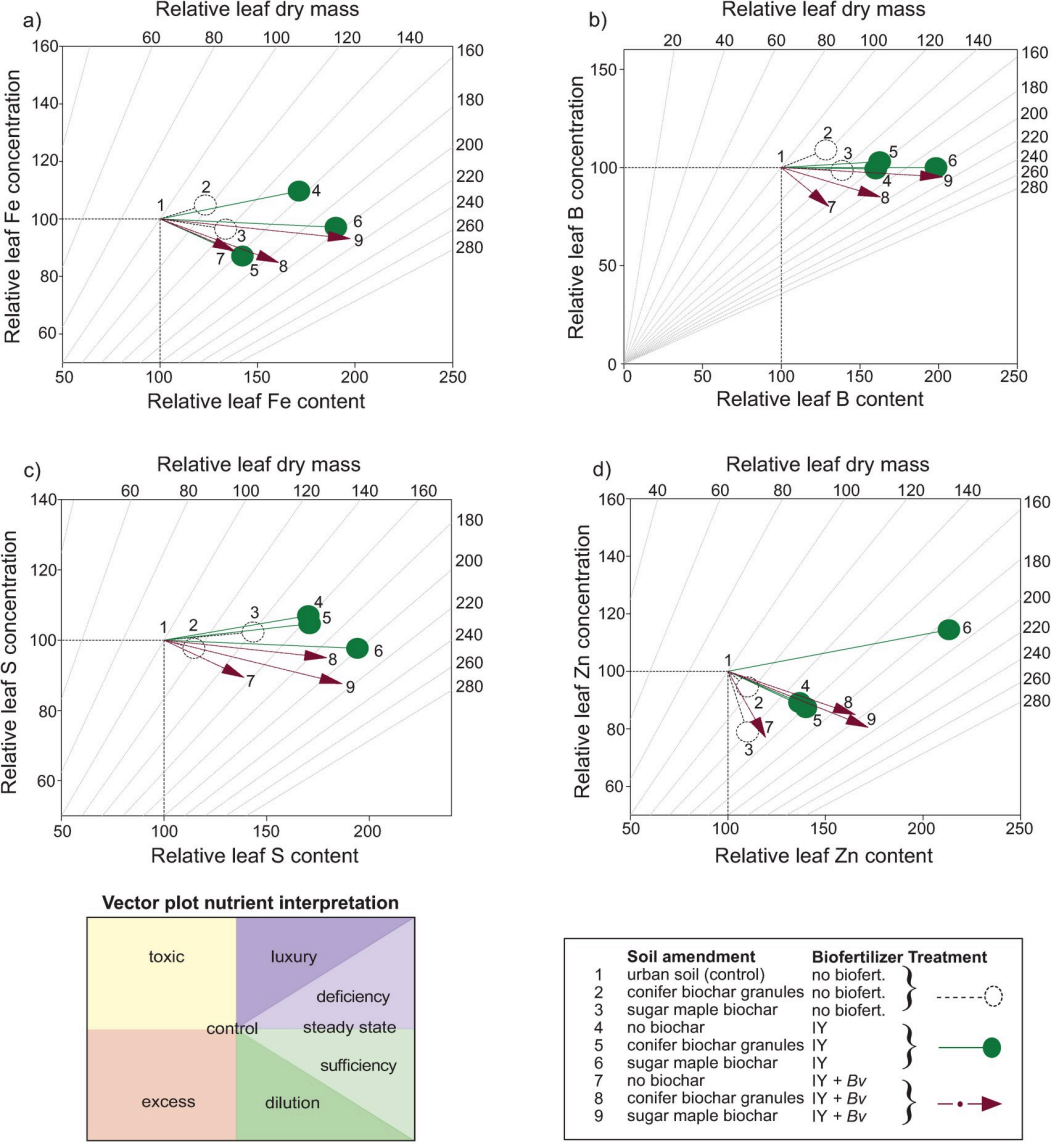
Vector diagrams of relative final leaf tissue micronutrient concentration vs content vs dry mass of: a) iron b) boron, c) sulphur, d) zinc. IY = inactivated yeast, *Bv* = *Bacillus velezensis*.

Vector analyses of micronutrients Fe, B, and S showed that sugar maple biochar with either IY alone or IY with Bv were generally the best treatments for foliar uptake of these nutrients (Figs 8 and 9). Foliar Zn vector analysis also showed that most treatments induced sufficiency in zinc, but sugar maple biochar with IY was a standout treatment where leaf biomass growth somewhat outstripped Zn availability (Figs 8D and 9D).

### Physiological responses

While final leaf nitrogen levels increased in treatments combining biochar and biofertilizers, leaf chlorophyll content index (CCI) levels were impacted by a spider mite outbreak during the final two weeks of the experiment. For mean leaf CCI values measured at the end of the experiment, both biochar and biofertilizers were significant main effects factors (Table 3), yet only the IY inoculation treatment was significantly different than control with a 28% higher mean (Dunnett p < 0.05) (S2C Fig in S1 File). Prior to the pest outbreak, a more pronounced difference in CCI measures among treatments with biofertilizers and biochars was recorded in week 10 (S2B Fig in S1 File, S4 Table in S1 File). Increases in CCI among biochar and biofertilizer treatments in week 10 ranged from +33% for sugar maple biochar with *Bv* + IY, up to +55% for IY alone. Final leaf chlorophyll fluorescence measures increased in treatments with biochars combined with biofertilizers (S2D Fig in S1 File) and biofertilizer had a significant effect on this variable (Table 3), but post-hoc comparisons to the control were not significant (Dunnett p < 0.05).

Conifer biochar granules with IY induced a significant positive WUE (water use efficiency) response (+29%) (S1 Table in S1 File, S3 Fig in S1 File). The biofertilizers had a significant influence over sapling leaf WUE and photosynthetic rate according to 2-way ANOVA (S2 & S3 Tables in S1 File), but neither biochar nor biofertilizer affected stomatal conductance, which was non-significant overall (S3 Table in S1 File). Mean photosynthetic rate and stomatal conductance rates for the treatments were not significantly different from the control (S3 Fig in S1 File).

## Discussion

As hypothesized, our results show that biochars and biofertilizers generally increase tree growth and macronutrient uptake in foliage, but the combined addition of bacterial and IY biofertilizers to pots with biochar yield far higher nutrient uptake and growth responses compared to biofertilizers or biochar alone. In several cases, growth after 3 months was doubled or tripled with combinations of biochar and biofertilizers relative to controls (Fig 2). Final sapling root fraction and leaf area ratios did not differ significantly among treatments and point to a balance of foliage and above and below ground growth across all treatments (Fig 3A and 3C).

Treatments with the largest positive responses in some cases were surprising. We expected that granulated conifer biochar combined with *Bv* and IY would yield the most favorable results in this urban soil due to an expected reduction in soil pH from this lower-pH biochar. Instead, the final soil pH increased significantly when the conifer biochar was applied alone (Fig 1A), and sugar maple biochar combined with inert IY without live *Bv* often resulted in equal or larger sapling growth increases (Fig 2). Prior published work led us to expect that treatments with IY+*Bv* would outperform IY by itself. *Bacillus polymyxa* combined with *S*. *cerevisiae* was shown to increase corn root and shoot growth compared to inoculation with just one of the microbes alone [95]. However, in most of our sapling growth and ecophysiological results, IY alone was comparable or even surpassed performance of combinations of IY with live *Bv*.

The enhanced sapling growth responses with IY may be explained by increases in N and key macronutrient availability and uptake by the saplings (Figs 4, 6 and 8). Another study comparing N fertilizer with *Bacillus*-based biofertilizer applications on wheat crops also concluded that the N fertilizer induced superior plant growth and plant nutrient uptake [96]. While few studies have examined the use of IY as a biofertilizer, Lonhienne et al. [85] also found that use of dead *S*. *cerevisiae* led to equal or greater increases in tomato and sugarcane growth, and N and P uptake, compared to inoculation with live yeast, and suggested that the yeast can provide a more bioavailable source of nutrients when dead than alive. Our results showed that IY by itself increased maple sapling biomass by 51%, leaf N by 94% and leaf P by 56% over the control (Fig 4A and 4B), which are values in line with or exceeding those of Lonhienne et al. Here, the application of IY by itself also significantly increased the mean leaf content of nutrients K, Mg, Ca, Cu, Al, B, Fe, Mo, Na, and S compared to the control (S6 Table in S2 File).

IY increased sapling uptake of important plant nutrients, and the combination with biochar appears to have generally enhanced the benefits to the saplings by further increasing growth and foliar nutrient concentrations. IY combined with sugar maple biochar provided the most impressive results by increasing sapling biomass by 83%, leaf N by 103% and leaf P by 91% compared to the control (Fig 4). Biochar has been known to generally increase P availability [97], and inactive yeast has been proven to increase soil P and P uptake in other crops [85]. Here, other plant macronutrients, such as K, Mg, Ca, etc. also increased significantly in leaves in IY treatments, and generally more so in biochar-biofertilizer treatment combinations (Fig 4). There is good evidence that yeasts and their associated fermentation by-products can increase availability of several important macro and micro-nutrients [98,99], but work is still preliminary and very little has been published on inert yeast and interactions between IY and live microbes in the soil. Except for copper, treatments in this trial combining biochar with biofertilizers also generally resulted in increased foliar contents of micronutrients (Fig 6). In the case of copper, exclusion at these levels is a benefit since this metal was approaching excess in treatments with biofertilizers (Fig 6 and S5 Fig in S1 File, S6 and S7 Tables in S2 File).

While the beneficial effects of IY on plant growth and nutrient uptake are convincing in results from this trial, our chlorophyll and photosynthesis results showed weaker yet still similar trends (S2 and S3 Fig in S1 File). We attribute these weaker trends in photosynthesis and chlorophyll results to a spider mite outbreak affecting chlorophyll content and photosynthetic activity in the final two weeks of this trial. All treatment combinations in week 10 of the experiment (just before the pest outbreak), showed significantly greater chlorophyll content in leaves with biochar and biofertilizer combinations (S2 Fig in S1 File), and photosynthesis and WUE increased in treatments with biofertilizers (S3 Fig in S1 File). While no prior published reports on the effects of IY on photosynthetic activity or leaf pigments could be found, foliar application of live *S*. *cerevisiae* significantly improved WUE in fava beans [100] and increased photosynthetic activity in strawberries [101], and soil inoculation with various live yeasts was shown to increase leaf chlorophyll in sugar beets [102].

Concerns have been expressed about the utility of higher pH biochar on high pH clay soils [103]. However, studies on calcareous soils have commonly found only slight effects of biochar on soil pH [104,105]. We hypothesized that biochar would help increase functionality in a disturbed anthropogenic soil, even if the soil is neutral to slightly alkaline. In our study, soil pH was moderated to levels similar to the control when biochars were combined with biofertilizers, suggesting that amending high-pH biochars with IY or *Bv* has potential to buffer liming effects of biochars. Despite some significant increases in pH among biochar and biofertilizer treatments, final soil pH was within a range of 6.88–7.19 across all treatments, which is close to neutral, and ideal for many plants. A soil acidification effect by IY has been noted in some other studies [106], but the addition of IY by itself did not lower final soil pH here. While the biofertilizers may influence substrate pH, the substrate pH can also influence *Bv* survival and pathogen-reducing surfactins [73], which could also affect tree growth outcomes in some conditions.

Biochars have been shown to generally increase and sustain soil microbial communities [107], and some combination of biochars and biofertilizers is likely to optimize plant responses [108]. In our study, large increases in tree growth were observed in soil amended with *Bacillus* and unbound sugar maple biochar, which was of a lower nutrient composition (Table 1) and smaller particle size than the conifer biochar granules. Our results are in line with Tao et al. [109] who found that biochar promoted the survival of *Bacillus subtilis*, and that survival of this microbe increased with smaller biochar particle sizes. While survival of PGPMs was not evaluated here, the sugar maple biochar may have created a suitable habitat for *Bv* and other beneficial soil microbes due to increased particle surface area of the smaller biochar particles [107,35], as well as the higher pH of the biochar from hardwood feedstock [73]. Biochar has been shown to increase soil microbial nitrogen fixation [110], and to reduce negative N-fixation responses commonly associated with N fertilization [111]. The high C:N ratio of the sugar maple biochar compared to the granulated conifer biochar (Table 1), and the resulting increase in C:N in all treatments with pure sugar maple biochar (Fig 1D and S1 Fig in S1 File), may have also stimulated additional diazotrophic N fixation in the soil microbial community and soil N retention of the biofertilizer nutrients. A similar response in increased N uptake in maize with increased nitrogen-fixing microbial activity was seen in another recent study combining biochar with *Enterobacter cloacae* and N fertilizer [112].

Based on prior published research indicating that biochars can help retain soil moisture [113], we expected that biochars with biofertilizers would increase soil moisture during drought, but no treatments had any significant effect on soil moisture after a dry-down period at the end of the experiment (Fig 1C). In our trial, 20 t/ha of biochar was selected as a near-optimal rate for general plant performance [43], but recent meta-analyses examining biochar effects on soil moisture indicate that biochar rates closer to 30 t/ha may be required to significantly influence soil moisture, and that biochar has a greater effect on soil moisture in coarser than finer soils [114,115]. The biochar amendment rate of 20 t/ha used in our experiment may not have been enough to strongly affect soil moisture, particularly in the medium- to fine-textured soil used here. Based on our measurements, soil moisture availability in dryer periods was not a major factor in the sapling growth effects observed. Slight decreases in final soil moisture in this trial could also be explained by the increased growth of the saplings in the same treatments and increased water uptake by the trees.

Understanding plant nutrient uptake pathways that result in beneficial plant responses in soils amended with biofertilizers and biochar requires more work [74,116]. Studies have shown that biochar plus PGPMs can improve soil quality and nutrient availability through activation of beneficial soil enzyme activity in tree plantations [117]. There is evidence that volatile organic compounds (VOCs) released by bacterial cells, including *Bv*, can induce increased growth in certain crops [118]. Other potential mechanisms for increased plant growth and health status with biochar and *Bacillus* biofertilizers include beneficial nutrient immobilization and release cycles triggered by microbial activity [74], pH and soil moisture changes [61], disease suppression by *Bv* [77,119], production of antibiotic lipopeptides by *Bv* [120,121], successful colonization by PGPMs of substrates via lipopeptide and biofilm production [121,122], and iron-chelating siderophore production in *Bv* [74].

Here, we observed that final soil N decreased nominally with the addition of sugar maple biochar (Fig 1D). However, this reduction is likely due to increased uptake of N by saplings with biochar and PGPM amendments, as reflected in increased tree biomass (Fig 2) and increased leaf N concentrations (Fig 4). All treatments with biofertilizers, particularly biofertilizers combined with biochar, showed increased foliar N content and correspondingly higher growth levels compared to the control as well as compared to treatments with just biochar (Fig 5). Foliar N concentrations measured in our study are somewhat lower than the reported average for *A*. *saccharinum* [123], but this may be attributed to a reduction of chlorophyll due to the spider mite outbreak in the final weeks of the experiment. Similar patterns of foliar N and growth in the two treatments combining sugar maple biochar with either IY or IY + *Bv* suggest that overall plant growth and health was maintained with these treatments despite the pest attack (Figs 4 and 5). Thus, we conclude that biochar-induced limitations on N availability were overcome here by combining biochar with IY and/or IY+*Bv*, and that the biochar dose rate of 20 t/ha, proposed as a median general dose to improve soil characteristics [42,43,124], was successful in helping to balance nutrient uptake, tree growth, and stress responses.

While our work was focused on examining biochar and biofertilizer effects on major plant nutrients and not on heavy metal remediation, some interesting observations emerged here related to copper. While Nkongolo et al. [125] classified *A*. *saccharinum* as a copper excluder and did not see evidence of much foliar uptake of Cu in a study testing heavy metal accumulation in this species, our results indicate that Cu was, in fact, accumulating at toxic levels in foliar tissues (Fig 7A). Average Cu foliar concentrations in silver maple grown in temperate climates have been reported as ~10 ppm in Cu-contaminated soil [125], or in the range of 4.46–5.79 ppm for *A*. *rubrum* x *A*. *saccharinum* (Freeman maple) hybrid in non-contaminated soil [126], compared to 16.33 ppm ±5.84 in the present study (S7 Table in S2 File). Mean Cu leaf concentration in the treatment with only conifer biochar granules was also significantly higher than in the other treatments (S7 Table in S2 File). Given that the conifer biochar contained 54.1 (±14.4) ppm Cu (Table 1), there is evidence that the Cu added through the granulated conifer biochar may have approached a level that inhibited plant growth. However, Cu accumulation in the foliage was significantly reduced in treatments that contained sugar maple biochar, biofertilizers, or combinations of biochar and biofertilizers (Fig 6A, S7 Table in S2 File). Some biochars have been reported to have significant potential for Cu immobilization in contaminated soil through reduction in plant root accumulation [127,128]. *Bacillus* and other beneficial soil bacteria have been reported to have tolerance to heavy metals [129], and to support Cu phytoremediation and/or bioabsorption in wastewater [130], and agronomic systems [131,132]. As for uptake in woody species, increases in foliar Cu uptake in apricot have also been reported with biofertilizer treatments [133].

## Conclusion

Our results indicate that the use of inactivated yeast and *Bacillus velezensis* as biofertilizers combined with biochars can substantially enhance tree performance. Both the unbound hardwood and granulated conifer biochars combined with either IY by itself, or added to live *B*. *velezensis* soil inoculation, significantly increased above- and below-ground growth and physiological performance in silver maple saplings grown in a low-quality disturbed urban soil. Further work is recommended to match biochar types, granulation, and biofertilizers to optimize results for specific soils and plant species. In the interest of future practical use and commercialization of biofertilized biochars, specific opportunities include: (1) exploration of the effect of various biochar fertilization and biofertilizers on native and natural biological communities; (2) comparison of inoculated biochars combined with different soil types and pH, particularly in nursery growing substrates and common urban anthropogenic soils; and (3) field trials in more seriously degraded and toxic urban soils to see whether biochar with *B*. *velezensis* and IY can increase plant health and survivability in brownfield sites.

## Supporting information

S1 File S1-S6 Figs and S1-S5 Tables showing additional soil, ecophysiological and nutrient response info and analyses are found in the supplementary document named Sifton et al. 2023 Biochar biofert. urb. forest. Supp. File1.
(PDF)

S2 File S6 and S7 Tables showing content and concentrations of sapling leaf nutrients are found in the supplementary document named Sifton et al. 2023 Biochar biofert. urb. forest. Supp. File2.
(PDF)

S3 File (DOCX)
