# Supplementary material for: Biochar-biofertilizer combinations enhance growth and nutrient uptake in silver maple grown in an urban soil
Source: PLoS One. 2023 Jul 18;18(7):e0288291. doi: 10.1371/journal.pone.0288291 (PMC10353828; doi:10.1371/journal.pone.0288291)
Supplement: S1 File — 2023 Biochar biofert. urb. forest. Supp. File1. (PDF) [file pone.0288291.s001.pdf]

Supplemental Tables and Figures for

Sifton MA, Smith SM, Thomas SC. 2023. Biochar-biofertilizer combinations enhance growth and nutrient uptake in silver maple grown in an urban soil. PLOS ONE. Accepted for publication June 27, 2023.

File Contents:

S1. Total final soil carbon
S2. Mean leaf chlorophyll content index at weeks a) 4, b) 10, c) 12; and d)leaf chlorophyll fluorescence at week 12
S3. Sapling gas exchange responses a) WUE, b) photosynthesis, c) stomatal conductance
S4. Final sapling leaf macronutrient concentration of a) nitrogen, b) phosphorus, c) potassium, d) magnesium, e) calcium.
S5. Final sapling leaf micronutrient concentration of a) copper, b) aluminum, c) manganese, d) molybdenum, e) sodium, f) zinc
S6. Final sapling leaf micronutrient concentration of a) iron, b) boron, c) sulphur
Table S1: A 1-way ANOVA table for vegetation and soil responses to biochar and biofertilizers treatments
Table S2: Mean sapling leaf physiological responses to biochar and biofertilizer treatments
Table S3: 2-way ANOVA results of treatment effects on sapling gas exchange
Table S4: Mean leaf chlorophyll content and chlorophyll fluorescence
Table S5: A 1-way ANOVA table of final sapling leaf nutrient content and concentration

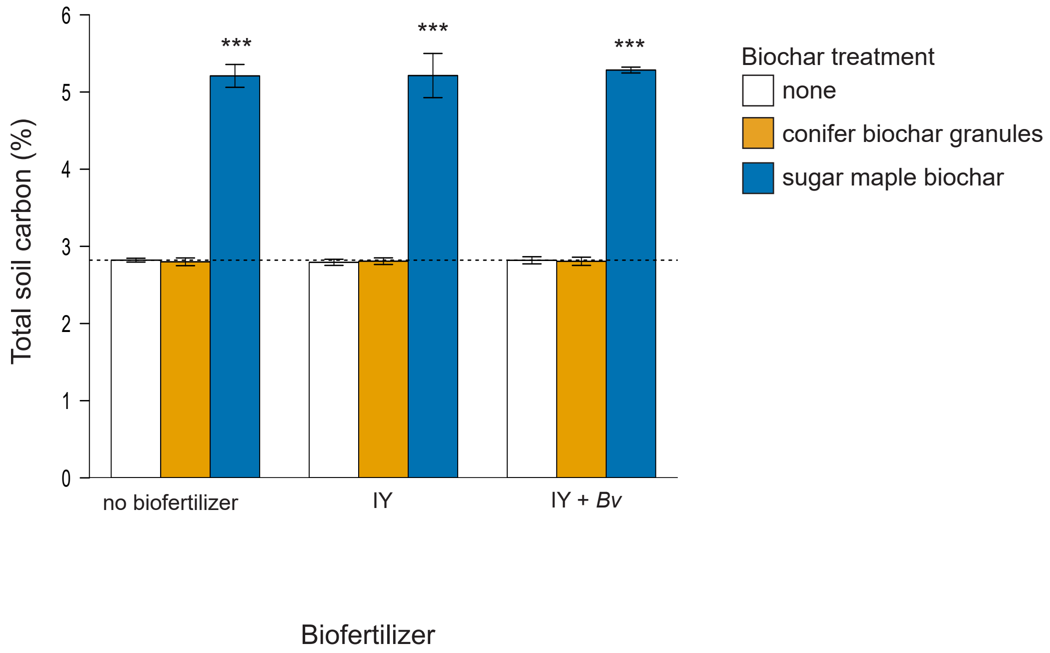

S1. Total soil carbon at the end of the 3-month experiment conducted from May-Aug, 2018 at the University of Toronto greenhouse. Statistically significant differences between control and treatment groups are indicated by asterisks (1-Way ANOVA, Dunnett's test, *** = $p < 0.001$). Dashed line indicates control mean. N= 13 trees for all treatments. Higher soil carbon in treatments with pure sugar maple biochar and lower carbon in granulated biochar treatments is as expected since a portion of the granulated char is comprised of a binder. IY = inactivated yeast, *Bv* = *Bacillus velezensis*.

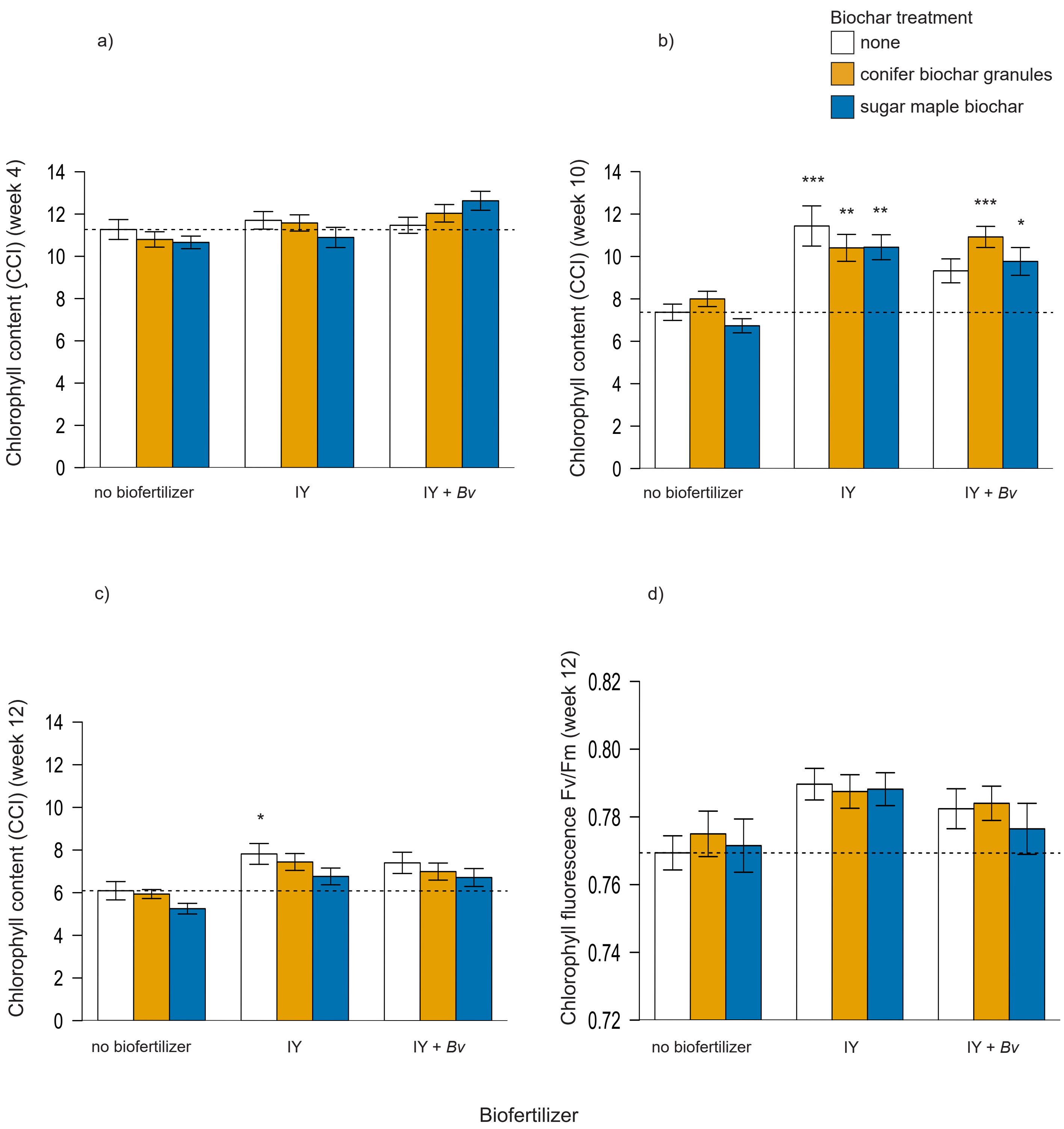

S2. Mean leaf chlorophyll content index, CCI, at: a) week 4, b) week 10, and c) week 12; leaf chlorophyll fluorescence (Fv/Fm) in week 12. Statistically significant differences between the control and the treatment groups are indicated by asterisk (1-way ANOVA, Dunnett's test, * = $p < 0.05$, ** = $p < 0.01$, *** = $p < 0.001$). Dashed line indicates control mean. CCI measurements were affected in the final 3 weeks of the experiment by an outbreak of spider mites, which feed on leaf sap and reduce chlorophyll content and leaf function. Leaf chlorophyll fluorescence, quantified as Fv/Fm, was also measured in weeks 10 and 12 but no significant effects were observed. IY = inactivated yeast, *Bv* = *Bacillus velezensis*.
Biochar treatments: □ = none, ■ = conifer biochar granules, ■ = sugar maple biochar.

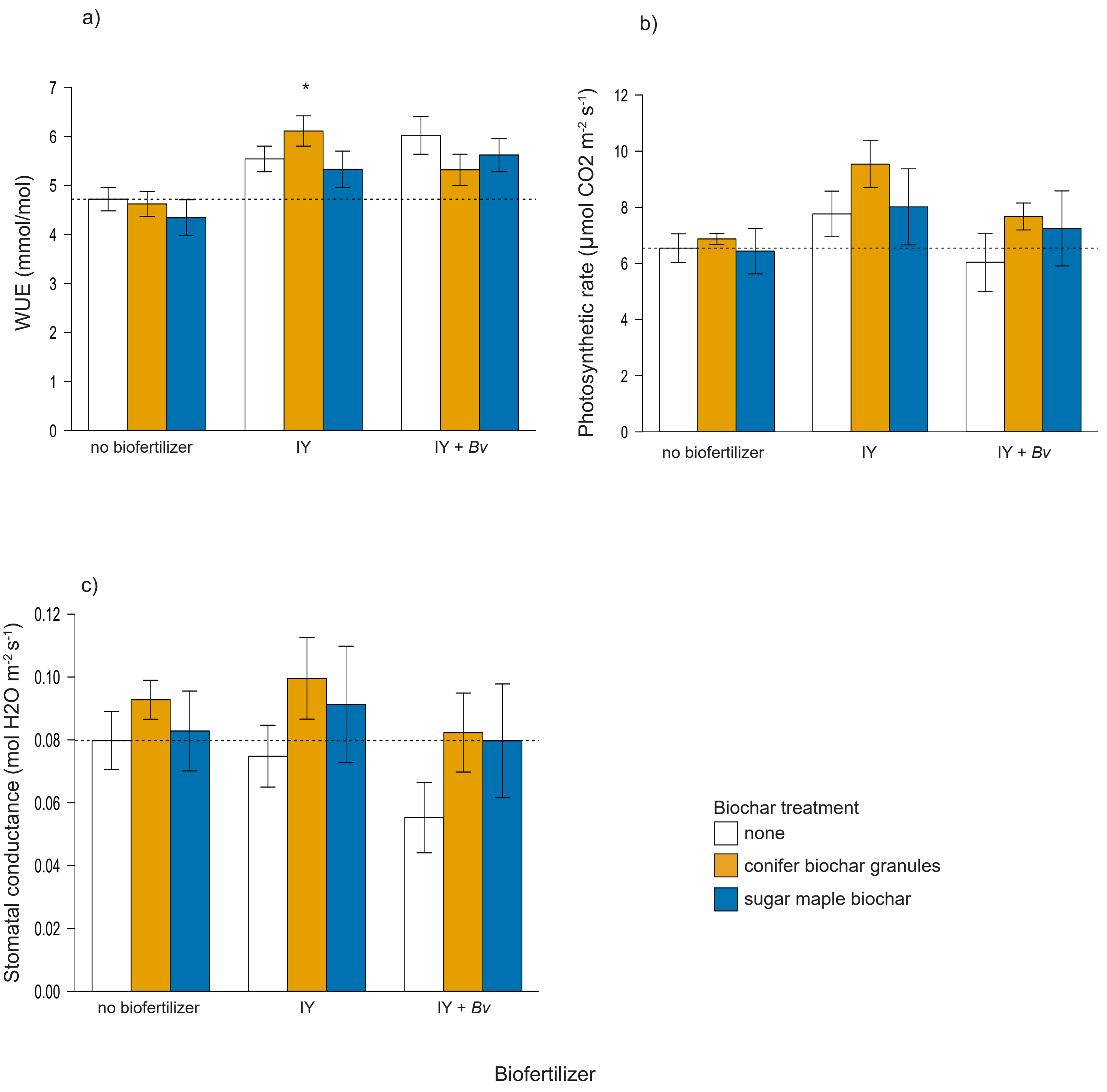

S3. Tree gas exchange responses a) Water Use Efficiency (WUE), b) A_{sat} photosynthetic rate (µmol CO2 m^{-2} s^{-1}), c) g_S stomatal conductance (mol H2O m^{-2} s^{-1}) at the end of the experiment. Dashed line indicates control mean.Statistically significant differences between the control and the treatment groups are indicated by and asterisk (1-way ANOVA, Dunnett's test, * = $p < 0.05$). 2-way ANOVA indicated that both biochar and clover biofertilizer were significant factors in WUE ($p < 0.05$). See supplementary tables 3 & 4 for further details. A spider mite outbreak in the final weeks of the experiment affected final results through reduction of leaf chlorophyll. IY = inactivated yeast, *Bv* = *Bacillus velezensis*.

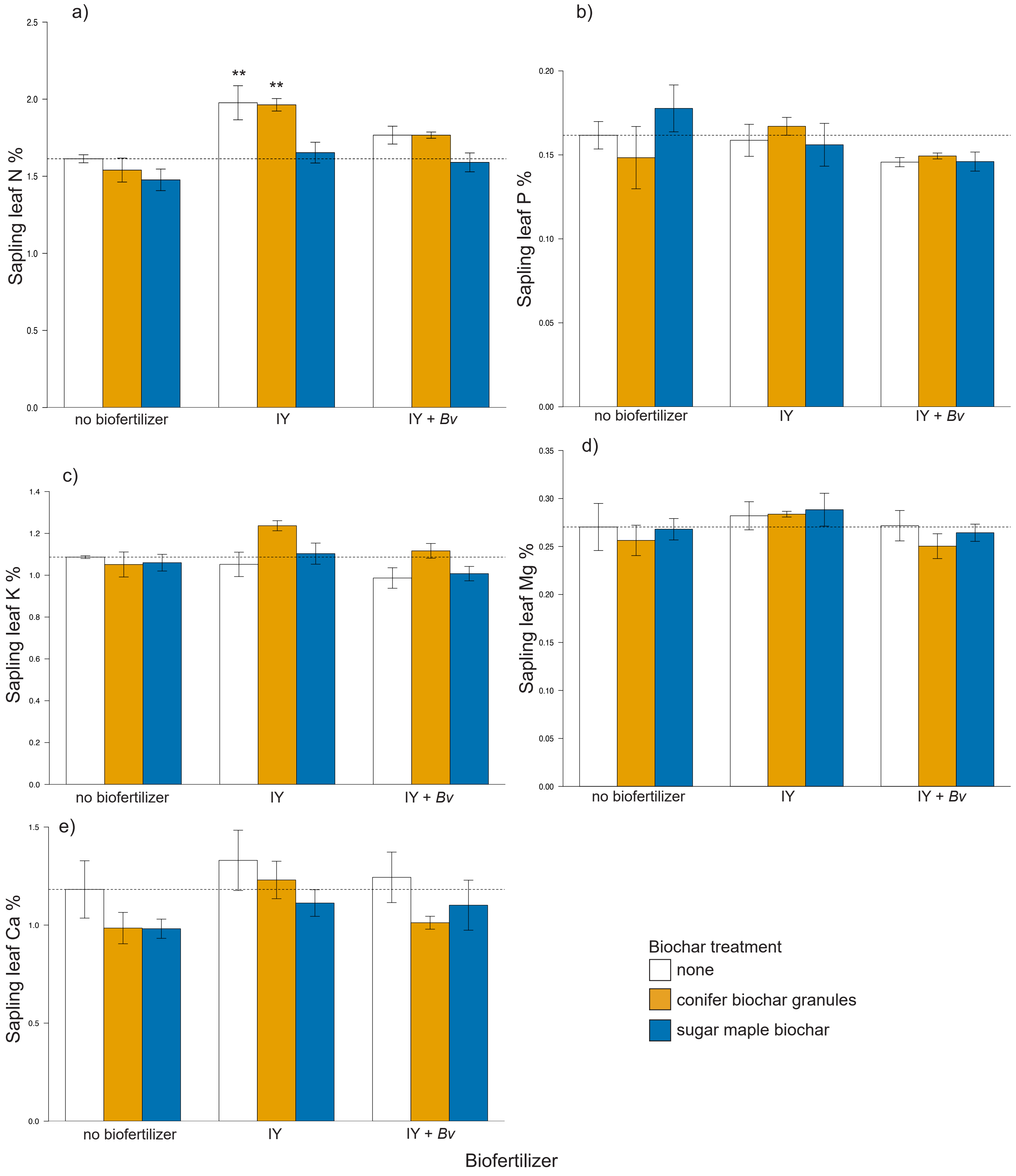

S4. Final sapling leaf macronutrient concentration (%) of: a) nitrogen, b) phosphorus, c) potassium, d) magnesium, e) calcium. Leaf sample tissues were dried at 60°C, ground, and subset into 3 reps each containing the same amount of materials from 4 replicates so that *n* = 3 for all results. Statistically significant differences between the control and the treatment groups are indicated by and asterisk (1-way ANOVA, Dunnett's test, ** = p < 0.01) Dashed line indicates control mean.
IY = inactivated yeast, *Bv* = *Bacillus velezensis*.

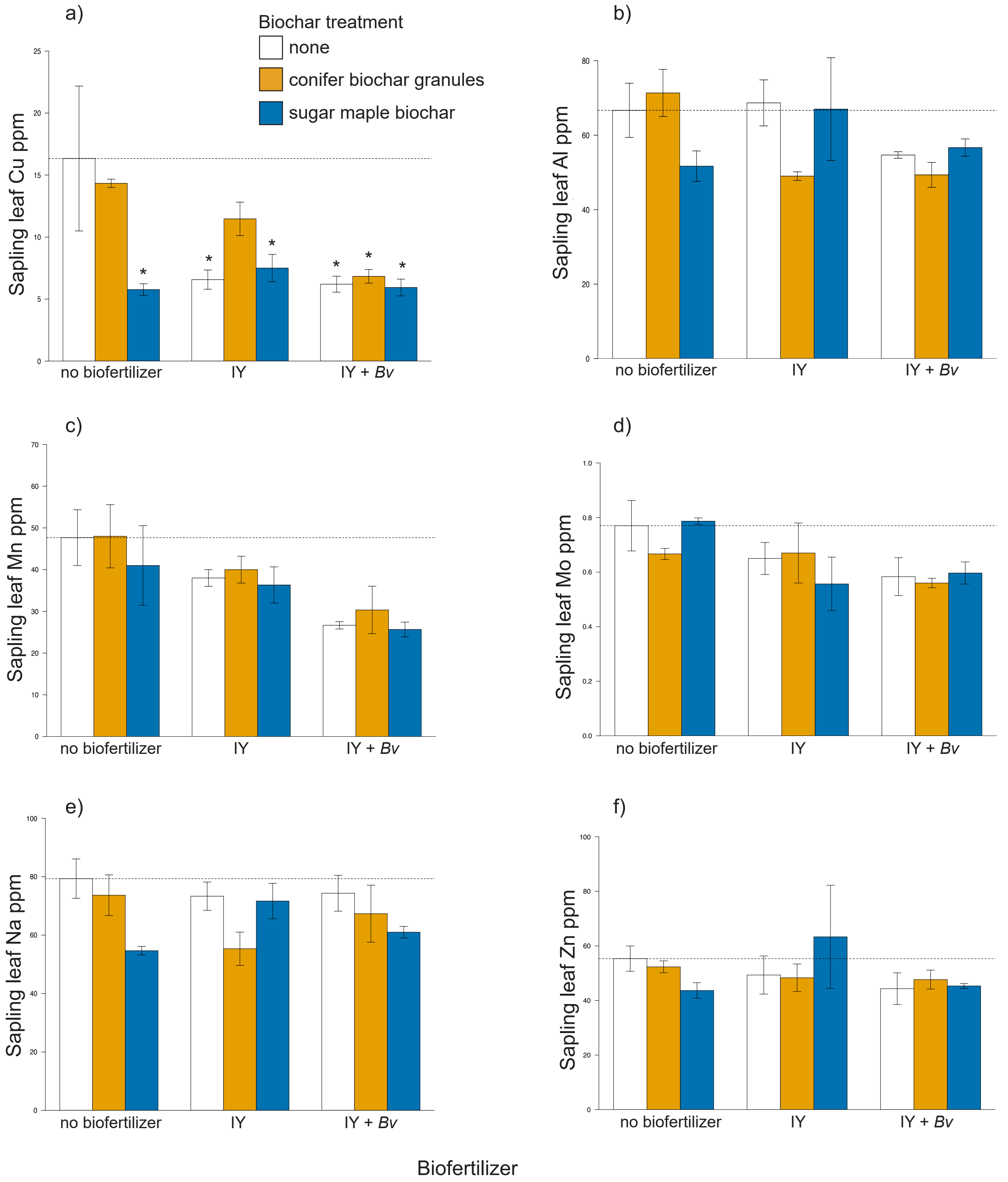

S5. Final sapling leaf micronutrient concentration (%) of: a) copper b) aluminum, c) manganese, d) molybdenum, e) sodium, f) zinc. Leaf sample tissues were dried at 60°C, ground, and subset into 3 reps each containing the same amount of materials from 4 replicates so that $n = 3$ for all results. Results from microwave acid digestion with ICP-OES (Agilent 5110 at AFL Labs, Guelph, ON, Canada). Statistically significant differences between the control and the treatment groups are indicated by asterisk (1-way ANOVA, Dunnett's test, * = $p < 0.05$) Dashed line indicates control mean. See Tables S5 and S6 1-way ANOVA results & values. IY = inactivated yeast, *Bv* = *Bacillus velezensis*.

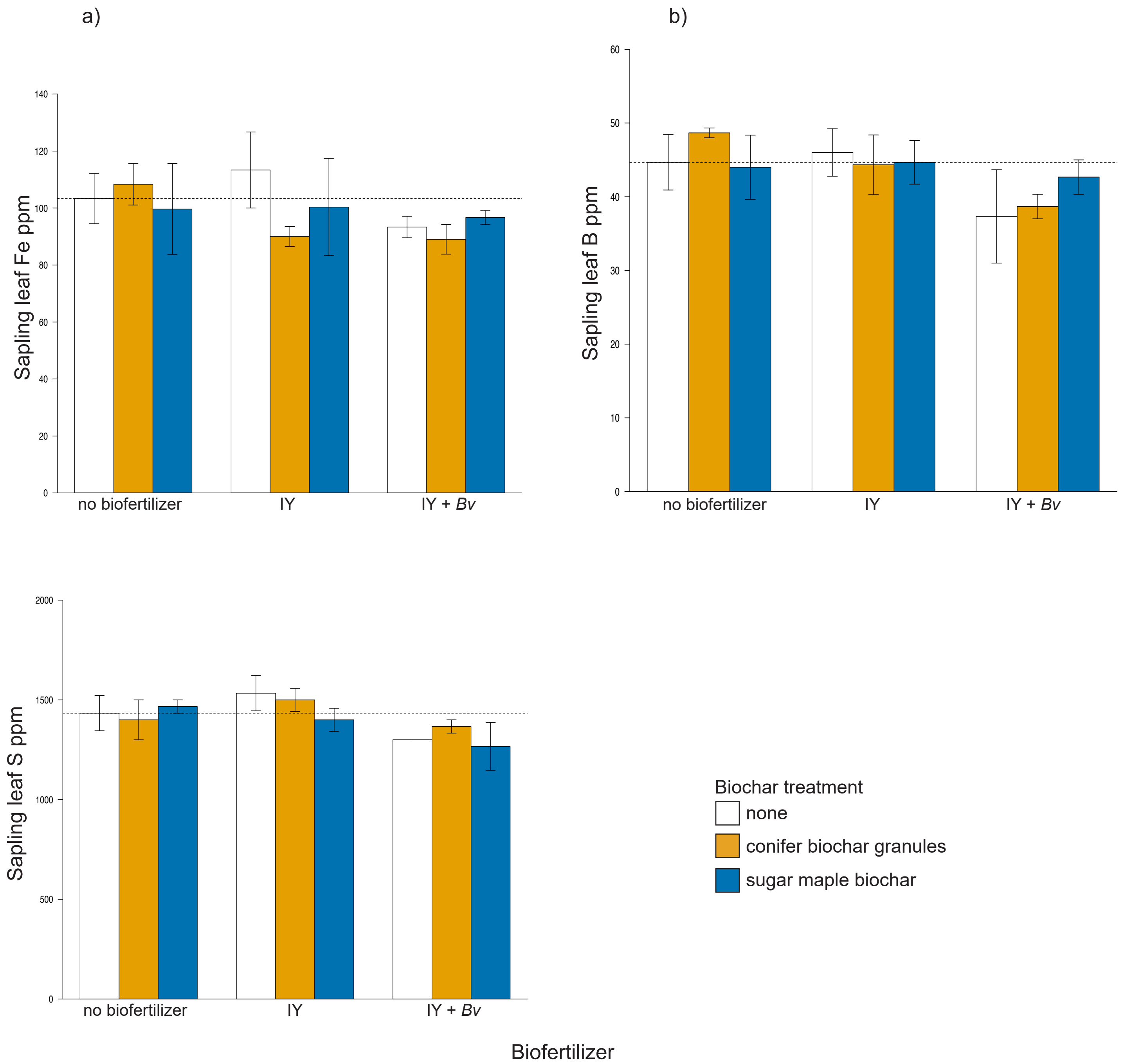

S6. Final sapling leaf micronutrient concentration (%) of: a) iron, b) boron, c) sulphur. Leaf sample tissues were dried at 60°C, ground, and subset into 3 reps each containing the same amount of materials from 4 replicates so that n = 3 for all results. Results from microwave acid digestion with ICP-OES (Agilent 5110 at AFL Labs, Guelph, ON, Canada). Treatments are not significantly different to the control at $p < 0.05$ according to Dunnett's post-hoc test. Dashed line indicates control mean. See Tables S5 and S7 for 1-way ANOVA results and values. IY = inactivated yeast, *Bv* = *Bacillus velezensis*.

Table S1. 1-way ANOVA table for vegetation and soil responses to biochar and biofertilizers.

Factor/effect	Effects	F-value ($DF_{between}$, DF_{within})	p-value
Sapling response	Total dry mass	6.918 (8, 108)	**< 0.001**
	Leaf area	11.820 (8, 108)	**< 0.001**
	Height	6.088 (8, 108)	**< 0.001**
	Caliper	1.689 (8, 108)	0.109
	Root fraction of biomass	1.345 (8, 108)	0.229
	Height:Caliper	5.093 (8, 108)	**< 0.001**
	Leaf area ratio	0.686 (8, 108)	0.703
	Leaf mass: leaf areas	1.509 (8, 108)	0.162
	Nitrogen mass in leaf tissues	15.09 (8, 108)	**< 0.001**
	Chlorophyll content in leaf tissues	4.303 (8, 108)	**< 0.001**
	Chlorophyll fluorescence in leaf tissues	1.621 (8, 108)	0.127
Soil response	pH	17.590 (8, 106)	**< 0.001**
	EC	7.104 (8, 107)	**< 0.001**
	Moisture	3.249 (8, 108)	**0.002**
	Total nitrogen	5.402 (8, 18)	**0.001**
	Total carbon	113.500 (8, 18)	**< 0.001**
Sapling gas exchange response	Leaf water use efficiency (WUE)	3.765 (8, 47)	**0.002**
	Photosynthetic rate (A_{sat})	1.664 (8, 47)	0.133
	Stomatal conductance (g_s)	0.893 (8, 36)	0.530

Significant results ($p < 0.05$) are bold.

Table S2. Mean (S.E.) physiological responses to biochar and biofertilizer treatments measured in sapling leaves.

Treatment	N	WUE % (mmol/mol)	A_{sat} (μmol CO_2 m^{-2} s^{-1})	g_s (mmol H2O m^{-2} s^{-1})
control	6	4.720 (0.238)	6.546 (0.509)	0.080 (0.009)
conifer biochar granules	5	4.622 (0.254)	6.873 (0.191)	0.093 (0.006)
conifer biochar granules + IY	7	**6.109** (0.309)	9.538 (0.832)	0.100 (0.013)
conifer biochar granules + IY + *Bv*	8	5.318 (0.319)	7.672 (0.480)	0.082 (0.013)
IY	8	5.540 (0.261)	7.763 (0.814)	0.075 (0.010)
sugar maple biochar	8	4.341 (0.365)	6.441 (0.810)	0.083 (0.013)
sugar maple biochar + IY	4	5.328 (0.372)	8.015 (1.355)	0.091 (0.019)
sugar maple biochar + IY + *Bv*	5	5.620 (0.339)	7.247 (1.336)	0.080 (0.018)
IY + *Bv*	5	6.021 (0.385)	6.044 (1.033)	0.055 (0.011)

Abbreviations: N, number of replicates; A_{sat}, photosynthetic rate; g_s, stomatal conductance; WUE, water use efficiency calculated as A_{sat}/mean transpiration rate. Significant values compared to the control according to Dunnett's test are in bold, $p < 0.05$. IY = inactivated yeast, *Bv* = *Bacillus velezensis*.

Table S3. 2-way ANOVA results of treatment effects on sapling gas exchange.

	Deg. Freedom	Sum of Squares	Mean Square	F Statistic	p-value
WUE					
Biochar	2	0.783	0.391	0.596	0.555
Biofertilizer	2	15.793	7.897	12.016	**<0.001**
Biochar x biofertilizer	4	3.216	0.804	1.223	0.314
Error	47	30.888	0.657		
A_{sat} photosynthetic rate					
Biochar	2	16.240	8.121	1.877	0.1643
Biofertilizer	2	35.890	17.946	4.148	**0.0219**
Biochar x biofertilizer	4	5.460	1.366	0.316	0.866
Error	47	203.340	4.326		
g_s stomatal conductance					
Biochar	2	0.005	0.002	2.300	0.111
Biofertilizer	2	0.002	<0.001	0.900	0.413
Biochar x biofertilizer	4	<0.001	<0.001	0.186	0.945
Error	47	0.047	<0.001		

Significant results ($p < 0.05$) are in bold.

Table S4. Mean (S.E.) leaf chlorophyll content and chlorophyll fluorescence measured in sapling leaves.

Treatment	CCI Week 4	CCI Week 10	CCI Week 12	Fv/Fm Week 10	Fv/Fm Week 12
control	11.269 (.471)	7.369 (.385)	6.092 (.430)	0.763 (.008)	0.769 (.005)
conifer biochar granules	10.800 (.363)	8.000 (.361)	5.938 (.211)	0.781 (.005)	0.775 (.007)
conifer biochar granules + IY	11.585 (.384)	**10.412**** (.635)	7.446 (.396)	0.771 (.009)	0.788 (.005)
conifer biochar granules + IY + Bv	12.046 (.415)	**10.931***** (.499)	7.000 (.401)	0.767 (.006)	0.784 (.005)
IY	11.708 (.416)	**11.442***** (.947)	**7.823*** (.488)	0.769 (.010)	0.790 (.005)
sugar maple biochar	10.662 (.298)	6.735 (.333)	5.254 (.249)	0.765 (.009)	0.772 (.008)
sugar maple biochar + IY	10.900 (.478)	**10.442**** (.589)	6.769 (.393)	0.762 (.010)	0.788 (.005)
sugar maple biochar + IY + Bv	12.639 (.449)	**9.777*** (.655)	6.723 (.420)	0.776 (.011)	0.777 (.008)
IY + Bv	11.477 (.381)	9.331 (.565)	7.408 (.498)	0.766 (.006)	0.782 (.006)

N=13 trees for all treatments. Results of Dunnett's test for statistical significance compared to control are indicated in bold by * = $p < 0.05$, ** = $p < 0.01$, *** = $p < 0.001$. Abbreviations: CCI, chlorophyll content index; (Fv/Fm), chlorophyll fluorescence. Using 1-way ANOVA, CCI in week 4 was significant ($p = 0.020$), but no significant differences were observed compared to the control. CCI in week 10 was highly significant (F: 8.143, on 8 and 108 DF, $p < 0.001$) with IY alone and all combinations of inoculants with biochars significantly higher than control. A spider mite outbreak in the final 3 weeks of the experiment reduced chlorophyll content in sapling leaves and affected results as chlorophyll content values were reduced across all treatments by the mites. Thus, CCI in week 12 was highly significant overall according to 1-way ANOVA (F: 4.303 on 8 and 108 DF, $p < 0.001$), but only the IY treatment remained significantly higher than the control. Chlorophyll fluorescence in weeks 10 and 12 was not significant according to 1-way ANOVA analysis, though final week 12 2-way ANOVA results showed that inoculants were a significant influence on results (see Table 2). IY = inactivated yeast, Bv = Bacillus velezensis.

Table S5. A 1-way ANOVA table of final sapling leaf nutrient content and concentration responses.

Leaf nutrient	Content		Concentration	
	F-value ($DF_{between}$, DF_{within})	p-value	F-value ($DF_{between}$, DF_{within})	p-value
N	14.34 (8, 99)	**< 0.001**	7.559 (8, 18)	**< 0.001**
P	10.63 (8, 99)	**< 0.001**	1.131 (8, 18)	0.3895
K	10.33 (8, 99)	**< 0.001**	2.877 (8, 18)	**0.0298**
Mg	12.47 (8, 99)	**< 0.001**	0.722 (8, 18)	0.6709
Ca	10.72 (8, 99)	**< 0.001**	1.373 (8, 18)	0.2728
Cu	11.28 (8, 99)	**< 0.001**	3.694 (8, 18)	**0.0101**
Al	12.09 (8, 99)	**< 0.001**	2.012 (8, 18)	0.1040
B	8.756 (8, 99)	**< 0.001**	0.945 (8, 18)	0.5055
Fe	9.566 (8, 99)	**< 0.001**	0.655 (8, 18)	0.7228
Mn	3.281 (8, 99)	**0.002**	2.317 (8, 18)	0.0661
Mo	5.089 (8, 99)	**< 0.001**	1.586 (8, 18)	0.1977
Na	8.000 (8, 99)	**< 0.001**	2.177 (8, 18)	0.0813
S	9.417 (8, 99)	**< 0.001**	1.420 (8, 18)	0.2541
Zn	11.83 (8, 99)	**< 0.001**	0.690 (8, 18)	0.6954

Significant results ($p < 0.05$) are in bold.

NOTE: See Supplemental Tables S6, S7 in separate PDF document due to landscape orientation named "Sifton et al. 2023 Biochar biofert. urb. forest. Supp. File2"
