# Supplementary material for: Biochar-biofertilizer combinations enhance growth and nutrient uptake in silver maple grown in an urban soil
Source: PLoS One. 2023 Jul 18;18(7):e0288291. doi: 10.1371/journal.pone.0288291 (PMC10353828; doi:10.1371/journal.pone.0288291)
Supplement: S2 File — 2023 Biochar biofert. urb. forest. Supp. File2. (PDF) [file pone.0288291.s002.pdf]

Table S6: Final content of sapling leaf nutrients & Table S7: Final concentration of sapling leaf nutrients

Table S6. Content (mass in mg) of sapling leaf nutrients at the end of the experiment.

Leaf nutrient	Treatment								
	**1**	**2**	**3**	**4**	**5**	**6**	**7**	**8**	**9**
	control	conifer biochar granules	sugar maple biochar	IY	conifer biochar granules + IY	sugar maple biochar + IY	IY + *Bv*	conifer biochar granules + IY + *Bv*	sugar maple biochar + IY + *Bv*
N	87.83 (6.85)	98.12 (4.66)	112.06 (6.15)	**170.53(12.21)**	**173.99(10.40)**	**178.53(15.01)**	**141.85 (8.79)**	**174.50(11.61)**	**176.48 (8.00)**
P	8.76 (0.65)	**9.38 (0.56)**	**13.41 (0.66)**	**13.67 (0.97)**	**14.78 (0.86)**	**16.75 (1.42)**	11.66 (0.64)	**14.73 (0.94)**	**16.22 (0.76)**
K	59.29 (4.89)	67.00 (3.26)	80.31 (3.96)	**93.84 (9.12)**	**109.65 (6.45)**	**119.94(10.92)**	79.46 (5.37)	**110.59 (7.99)**	**111.93 (5.22)**
Mg	14.62 (1.16)	16.32 (0.78)	20.43 (1.26)	**24.34 (1.74)**	**25.18 (1.57)**	**31.08 (2.62)**	**21.80 (1.40)**	**24.57 (1.41)**	**29.31 (1.24)**
Ca	63.59 (5.00)	62.78 (3.41)	74.97 (4.90)	**113.45 (8.43)**	**108.69 (6.82)**	**120.78(11.23)**	**100.07 (7.76)**	**99.33 (5.54)**	**122.35 (8.12)**
Cu	0.085 (0.012)	0.092 (0.005)	**0.044 (0.002)**	**0.055 (0.003)**	0.100 (0.006)	0.080 (0.008)	**0.049 (0.003)**	0.067 (0.003)	0.066 (0.005)
Al	0.361 (0.030)	0.453 (0.023)	0.391 (0.021)	**0.586 (0.038)**	0.434 (0.024)	**0.689 (0.057)**	0.438 (0.024)	0.484 (0.029)	**0.629 (0.030)**
B	0.243 (0.020)	0.312 (0.016)	0.337 (0.026)	**0.395 (0.027)**	**0.389 (0.021)**	**0.482 (0.042)**	0.304 (0.031)	**0.380 (0.023)**	**0.475 (0.025)**
Fe	0.559 (0.044)	0.689 (0.033)	0.747 (0.050)	**0.958 (0.059)**	**0.795 (0.044)**	**1.064 (0.106)**	0.745 (0.040)	**0.870 (0.045)**	**1.073 (0.048)**
Mn	0.254 (0.018)	0.305 (0.023)	0.318 (0.040)	0.339 (0.033)	0.357 (0.029)	**0.404 (0.050)**	0.213 (0.012)	0.305 (0.037)	0.285 (0.015)
Mo	0.004(<0.001)	0.004(<0.001)	**0.006(<0.001)**	**0.006(<0.001)**	**0.006(<0.001)**	**0.006(<0.001)**	0.005(<0.001)	0.006(<0.001)	**0.007(<0.001)**
Na	0.427 (0.029)	0.469 (0.026)	0.415 (0.023)	**0.651 (0.063)**	0.491 (0.039)	**0.793 (0.089)**	0.594 (0.038)	**0.651 (0.038)**	**0.675 (0.026)**
S	7.774 (0.608)	8.904 (0.438)	**11.144(0.594)**	**13.228(0.951)**	**13.278(0.786)**	**15.092(1.244)**	10.422(0.600)	**13.508(0.921)**	**14.100(0.885)**
Zn	0.298 (0.020)	0.334 (0.016)	0.331 (0.018)	0.414 (0.025)	**0.424 (0.023)**	**0.637 (0.063)**	0.351 (0.023)	**0.476 (0.042)**	**0.503 (0.021)**

Leaf nutrients were calculated using mean leaf nutrient concentration values multiplied by total dry leaf mass for each replicate (n = 12 for all treatments). Results of Dunnett's test for statistical significance compared to control are indicated in bold where p < 0.05. IY = inactivated yeast, *Bv* = *Bacillus velezensis*.

Table S7. Concentration of maple sapling leaf nutrients at the end of the experiment.

Leaf nutrient	Treatment								
	**1**	**2**	**3**	**4**	**5**	**6**	**7**	**8**	**9**
	control	conifer biochar granules	sugar maple biochar	IY	conifer biochar granules + IY	sugar maple biochar + IY	IY + *Bv*	conifer biochar granules + IY + *Bv*	sugar maple biochar + IY + *Bv*
N %	1.613 (0.026)	1.540 (0.078)	1.477 (0.070)	**1.977 (0.111)**	**1.963 (0.041)**	1.653 (0.067)	1.767 (0.058)	1.767 (0.020)	1.590 (0.061)
P %	0.162 (0.008)	0.148 (0.019)	0.178 (0.014)	0.159 (0.010)	0.167 (0.005)	0.156 (0.013)	0.146 (0.003)	0.149 (0.002)	0.146 (0.006)
K %	1.087 (0.007)	1.051 (0.060)	1.060 (0.040)	1.052 (0.058)	1.237 (0.024)	1.103 (0.050)	0.987 (0.049)	1.117 (0.035)	1.008 (0.034)
Mg %	0.270 (0.025)	0.256 (0.016)	0.268 (0.011)	0.282 (0.015)	0.284 (0.003)	0.288 (0.017)	0.272 (0.016)	0.250 (0.013)	0.264 (0.009)
Ca %	1.182 (0.146)	0.985 (0.080)	0.981 (0.049)	1.330 (0.154)	1.230 (0.095)	1.112 (0.068)	1.243 (0.129)	1.012 (0.033)	1.101 (0.128)
Cu ppm	16.33 (5.84)	14.33 (0.33)	**5.77 (0.47)**	**6.57 (0.78)**	11.47 (1.35)	**7.50 (1.10)**	**6.20 (0.64)**	**6.83 (0.55)**	**5.93 (0.68)**
Al ppm	66.67 (7.26)	71.33 (6.33)	51.67 (4.10)	68.67 (6.17)	49.00 1.15	67.00 (13.80)	54.67 (0.88)	49.33 (3.33)	56.67 (2.33)
B ppm	44.67 (3.76)	48.67 (0.67)	44.00 (4.36)	46.00 (3.21)	44.33 (4.06)	44.67 (2.96)	37.33 (6.33)	38.67 (1.67)	42.67 (2.33)
Fe ppm	103.33 (8.82)	108.33 (7.26)	99.67 (15.94)	113.33 (13.33)	90.00 (3.51)	100.33 (17.03)	93.33 (3.76)	89.00 (5.20)	96.67 (2.40)
Mn ppm	47.67 (6.69)	48.00 (7.57)	41.00 (9.54)	38.00 (2.00)	40.00 (3.21)	36.33 (4.33)	26.67 (0.88)	30.33 (5.70)	25.67 (1.76)
Mo ppm	0.77 (0.09)	0.67 (0.02)	0.79 (0.01)	0.65 (0.06)	0.67 (0.11)	0.56 (0.10)	0.58 (0.07)	0.56 (0.02)	0.60 (0.04)
Na ppm	79.33 (6.74)	73.67 (6.96)	54.67 (1.45)	73.33 (4.84)	55.33 (5.70)	71.67 (6.06)	74.33 (6.12)	67.33 (9.77)	61.00 (2.00)
S ppm	1433 (88)	1400 (100)	1467 (33)	1533 (88)	1500 (58)	1400 (58)	1300 (0)	1367 (33)	1267 (120)
Zn ppm	55.33 (4.631)	52.33 (2.186)	43.67 (2.848)	49.33 (6.984)	48.33 (5.044)	63.33 (18.91)	44.33 (5.783)	47.67 (3.480)	45.33 (0.882)

Leaf nutrients were analyzed using the following methods: C: combustion elemental analysis (CN628 LECO, LECO Instruments Ltd., Mississauga, ON, Canada); N combustion and thermal conductivity detection (Elementar Vario Macro Cube, at AFL, Guelph, ON, Canada); Al, B, Ca, Cu, Fe, Mg, Mn, Mo, P, K, Na, Zn: microwave acid digestion with ICP-OES (Agilent 5110, at AFL, Guelph, ON, Canada)). Values are means (±SE) of triplicate measurements of samples that were evenly-pooled from dried, ground leaf tissue. Results of Dunnett's test for statistical significance compared to control are indicated in bold where $p < 0.05$. IY = inactivated yeast, *Bv* = *Bacillus velezensis*.

NOTE: See Supplemental Tables S1-S6 & Tables S1-S5 in separate PDF document due to landscape orientation named “Sifton et al. 2023 Biochar biofert. urb. forest. Supp. File1”.
