# Supplementary material for: Biochar-biofertilizer combinations enhance growth and nutrient uptake in silver maple grown in an urban soil
Source: PLoS One. 2023 Jul 18;18(7):e0288291. doi: 10.1371/journal.pone.0288291 (PMC10353828; doi:10.1371/journal.pone.0288291)
Supplement: S3 File — (DOCX) [file pone.0288291.s003.docx]

Data for this article can be found here in the Borealis Canadian Dataverse’’- Repository: <https://doi.org/10.5683/SP3/H5U4OO>
